# Supplementary material for: Phase II randomised control feasibility trial of a nutrition and physical activity intervention after radical prostatectomy for prostate cancer
Source: BMJ Open. 2019 Nov 6;9(11):e029480. doi: 10.1136/bmjopen-2019-029480 (PMC6858112; doi:10.1136/bmjopen-2019-029480)
Supplement: Author's manuscript [file bmjopen-2019-029480.draft_revisions.pdf]

BMJ Open is committed to open peer review. As part of this commitment we make the peer review history of every article we publish publicly available.

When an article is published we post the peer reviewers' comments and the authors' responses online. We also post the versions of the paper that were used during peer review. These are the versions that the peer review comments apply to.

The versions of the paper that follow are the versions that were submitted during the peer review process. They are not the versions of record or the final published versions. They should not be cited or distributed as the published version of this manuscript.

BMJ Open is an open access journal and the full, final, typeset and author-corrected version of record of the manuscript is available on our site with no access controls, subscription charges or pay-per-view fees (http://bmjopen.bmj.com).

If you have any questions on BMJ Open's open peer review process please email info.bmjopen@bmj.com

BMJ Open

PHASE II RANDOMISED CONTROL TRIAL OF A NUTRITION AND PHYSICAL ACTIVITY INTERVENTION AFTER RADICAL PROSTATECTOMY FOR PROSTATE CANCER

Journal:	*BMJ Open*
Manuscript ID	bmjopen-2019-029480
Article Type:	Research
Date Submitted by the Author:	28-Jan-2019

Complete List of Authors:	Hackshaw-McGeagh, Lucy E.; University of Bristol, NIHR Biomedical Research Centre<br>Penfold, Chris; University of Bristol, Musculoskeletal Research Unit<br>Shingler, Ellie; University of Bristol, NIHR Biomedical Research Centre<br>Robles, Luke; University of Bristol, Bristol Medical School: Population Health Sciences; University of Bristol, MRC Integrative Epidemiology Unit<br>Perks, Claire; University of Bristol, Bristol Medical School: Translational Health Sciences<br>Holly, Jeff; University of Bristol, Translational Health Sciences<br>Rowe, Edward; North Bristol NHS Trust, Urology<br>Koupparis, Anthony; North Bristol NHS Trust, Urology<br>Bahl, Amit; University Hospitals Bristol, Bristol Haematology and Oncology Centre<br>Persad, Raj; North Bristol NHS Trust<br>Shiridzinomwa, Constance; North Bristol NHS Trust, Clinical Research Centre<br>Johnson, Lyndsey; North Bristol NHS Trust, Clinical Research Centre<br>Biernacka, Kalina; University of Bristol, Translational Health Sciences<br>Frankow, Aleksandra; University of Bristol, Translational Health Sciences<br>Woodside, Jayne; Queens University Belfast, Institute for Global Food Security<br>Gilchrist, Sarah; Queens University Belfast, Institute for Global Food Security<br>Oxley, Jon; North Bristol NHS Trust, Department of Cellular Pathology<br>Abrams, Paul; Bristol Urological Institute, Southmead Hospital<br>Lane, Athene; University of Bristol, School of Social and Community Medicine; University of Bristol, NIHR Biomedical Research Centre<br>Martin, Richard; University of Bristol, Social Medicine; University of Bristol, NIHR Biomedical Research Centre

Keywords:	Prostate disease < UROLOGY, UROLOGY, Clinical trials < THERAPEUTICS, SURGERY, PREVENTIVE MEDICINE, Urological tumours < ONCOLOGY

SCHOLARONE™
Manuscripts

PHASE II RANDOMISED CONTROL TRIAL OF A NUTRITION AND PHYSICAL ACTIVITY INTERVENTION AFTER RADICAL PROSTATECTOMY FOR PROSTATE CANCER

L Hackshaw-McGeagh[1], C Penfold[2], E Shingler[1*], L A Robles[3,4*], C M. Perks[5], J M. P. Holly[5], E Rowe[6], A Koupparis[6], A Bahl[6], R Persad[6], C Shiridzinomwa[7], L Johnson[7], K M Biernacka[5], A Frankow[5], J V Woodside[8], S Gilchrist[8], J Oxley[9], P Abrams[6], J. A Lane[1,10,11*], R M Martin[1,4,10*]

Affiliation of author

[1] National Institute for Health Research (NIHR) Bristol Biomedical Research Centre (Nutrition Theme), Level 3, University Hospitals Bristol Education Centre, Upper Maudlin Street, Bristol, BS2 8AE

[2] National Institute for Health Research (NIHR) Bristol Biomedical Research Centre (Surgical Innovation Theme), Translational Health Sciences, Musculoskeletal Research Unit, University of Bristol, Level 2 Learning and Research Building, Southmead Hospital, Bristol, BS10 5NB

[3] Bristol Medical School: Population Health Sciences, University of Bristol, Oakfield House, Oakfield Grove, Clifton BS8 2BN

[4] MRC Integrative Epidemiology Unit, University of Bristol, Canynge Hall, 39 Whatley Road, Bristol, BS8 2PS

[5] Bristol Medical School: Translational Health Sciences, IGFs and Metabolic Endocrinology Group, University of Bristol, Level 2 Learning & Research Building, Southmead Hospital, Bristol, BS10 5NB

[6] Bristol Urological Institute, Level 3 Learning and Research Building, Southmead Hospital, Bristol, BS10 5NB

[7] Clinical Research Centre, North Bristol NHS Trust, Southmead Hospital, BS10 5NB

[8] Nutrition Group, Institute for Global Food Security (Centre for Public Health), School of Medicine, Dentistry and Biomedical Sciences, Queen's University Belfast, Institute of Clinical Science B (First Floor), Grosvenor Road, Belfast, BT12 6BJ

[9] Department of Cellular Pathology, Pathology Sciences Building, North Bristol NHS Trust, Southmead Hospital, Bristol, BS10 5NB

For peer review only - http://bmjopen.bmj.com/site/about/guidelines.xhtml

For peer review only

[10] Bristol Medical School: Population Health Sciences, University of Bristol, Canynge Hall, 39 Whatley Road, Bristol, BS8 2PS

[11] Bristol Randomised Trials Collaboration (BRTC), Bristol Medical School: Population Health Sciences, University of Bristol, Canynge Hall, 39 Whatley Road, Bristol, BS8 2PS

* These authors contributed equally to this work

Corresponding author

Dr JA Lane, Bristol Medical School: Population Health Sciences, University of Bristol, Canynge Hall, 39 Whatley Road, Bristol, BS8 2PS, 0117 928 7335, Athene.Lane@bristol.ac.uk

Word count: 4177

Tables and figures: 6

For peer review only

Abstract

Objective: Dietary factors and physical activity may alter prostate cancer progression. We explored the feasibility of lifestyle interventions following radical prostatectomy for localised prostate cancer.

Design: Patients were recruited into a pre-surgical observational cohort; following radical prostatectomy, they were offered randomisation into a 2x3 factorial randomised controlled trial (RCT).

Setting: A single National Health Service trust in the South West of England, UK.

Participants: Those with localised prostate cancer and listed for radical prostatectomy were invited to participate.

Randomisation: Random allocation was performed by the Bristol Randomised Trial Collaboration via an online system.

Interventions: Men were randomised into both a modified nutrition group (either increased vegetable and fruit, and reduced dairy milk; or lycopene supplementation; or control) and a physical activity group (brisk walking or control), for six months.

Blinding: Only the trial statistician was blind to allocations.

Primary outcome measures: Collected at trial baseline, three and six months, with daily adherence reported throughout. Primary outcomes were measures of feasibility: randomisation rates and intervention adherence at six months.

Results: 108 men entered the pre-surgical cohort and 81 were randomised into the post-surgical RCT (randomisation rate: 93.1%), 75 completed the trial. Of 25 men in the nutrition intervention, 10 (40.0%; 95% CI: 23.4%-59.3%) adhered to the fruit and vegetable recommendations and 18 (72.0% 95% CI 52.4%-85.7%) to reduced dairy intake. Adherence to lycopene (n=28), was 78.6% (95% CI: 60.5%-89.8%), whilst 21/39 adhered to the walking intervention (53.8%; 95% CI: 38.6%-68.4%). Most men were followed up at six months (75/81; 92.6%)**.** Three “possibly related” adverse events were indigestion, abdominal bloating and knee pain.

For peer review only - http://bmjopen.bmj.com/site/about/guidelines.xhtml

Conclusions: Interventions were deemed feasible, with high randomisation rates and good adherence. A definitive RCT is proposed.

Trial registration: ISRCTN 99048944

Funding: NIHR Biomedical Research Centre at University Hospitals Bristol NHS Foundation Trust and the University of Bristol.

Strengths And Limitations Of This Study

- Robust, gold standard, randomised control trial 2x3 design, with 6 month follow up
- Excellent retention rates, with only 6 participants withdrawing or being lost to follow up
- Outcomes include objective biochemical measures which are not open to responder bias
- Except for the statistician, all researchers and participants were unblinded; blinding was not feasible given the nature of the intervention.

For peer review only

INTRODUCTION

Prostate cancer is the most common male cancer in the UK. There were 43 400 new cases diagnosed in 2012 [1], accounting for 25% of UK male cancers. Furthermore, in 2014 there were 11 300 prostate cancer deaths, making the disease the second commonest cause of UK cancer-related death [1]. Following improvements in clinical diagnosis and treatment leading to rising survival rates, more men are living longer with the disease and there is growing interest in developing lifestyle modifications for tertiary prevention of prostate cancer morbidity and mortality [2].

Evidence summarised by the World Cancer Research Fund [3] suggests that vegetables and fruit, and associated micronutrients (e.g. carotenoids, some vitamins, and flavonoids), may reduce prostate cancer risk, whilst calcium and dairy intake may increase risk. Our recent review of 44 randomised controlled trials (RCTs) of dietary, nutrition and physical activity interventions suggest various dietary interventions (e.g. low fat diet, soya and soya foods, selenium, lycopene or green tea) may reduce prostate cancer progression and mortality [2]. However, many studies were assessed as being of low quality (with high or unclear risk of bias), underpowered, inadequately reported, of short duration or measuring surrogate outcomes, such as prostate specific antigen doubling time which has an uncertain relationship with prostate cancer progression.

Lycopene, an antioxidant phytonutrient, has been observed to reduce prostate cancer progression [4, 5], while high-fat milk intake has been positively associated with prostate cancer progression [6]. However, robust RCT evidence for the effects of lycopene or milk intake in relation to prostate cancer progression is lacking [3].

Moderate-vigorous physical activity is another potentially promising intervention [7], but the evidence is also largely based on observational data: RCTs are needed for definitive evidence. Moderate–vigorous intensity activity is defined as activities that cause sweating and increased heart

For peer review only - http://bmjopen.bmj.com/site/about/guidelines.xhtml

and respiratory rate, of which brisk walking is an example [8]. Brisk walking has been associated with anti-cancer cellular behaviour [9], and does not require specialised equipment or training.

Some men spontaneously and positively alter lifestyle behaviours on receiving a diagnosis of prostate cancer. However, for many men this is not the case [10-12] and additional interventions are necessary to promote and maintain behavioural change [13-15]. We established a 2 x 3 factorial RCT - the Prostate Cancer: Evidence of Exercise and Nutrition Trial (PrEvENT) - to explore the feasibility of introducing modified nutrition (increased vegetables and fruit combined with reduced dairy milk) or lycopene supplements and physical activity (brisk walking) interventions in men treated with radical prostatectomy for localised prostate cancer. Here we report the primary trial findings.

MATERIALS AND METHODS

Trial Design and Participants

The trial protocol has been published elsewhere [16]. In brief, men from a single National Health Service (NHS) trust in the South West of England, UK, who were diagnosed with localised prostate cancer and listed for radical prostatectomy were invited to participate in PrEvENT between August 2014 and May 2016. There were two phases: (i) recruitment of men scheduled for radical prostatectomy into a baseline cohort for epidemiological characterisation (i.e. pre-surgical questionnaire data and blood sampling) of the target population; and ii) recruitment of the men once they had their radical prostatectomy into a 2x3 factorial RCT.

Cohort and RCT inclusion criteria were: localised prostate cancer; undergoing radical prostatectomy; capacity to provide informed consent; aged 18 or over; and sufficient understanding of the English language. Exclusion criteria were: inability to give informed consent; unavailability for follow-up; identified as unsuitable to participate by the treating clinician; co-morbidities, allergies or religious

For peer review only

beliefs which could prevent participation in the intervention (RCT only); regularly taking lycopene supplements or routinely exercising vigorously (RCT only).

Nurse-led consent at cohort recruitment included optional approval for prostate tissue extracted during surgery to be used for research purposes and willingness to be contacted about future involvement in further research. Approximately six weeks after surgery, the men who were participating in the cohort were invited to participate in the RCT.

Randomisation

Random allocation was performed by the Bristol Randomised Trial Collaboration (BRTC) via an online system to ensure that the recruiting nurses could not uncover allocation of trial group in advance (concealment of allocation). The research nurse, research team and men were not blind to intervention allocation because this would have been unfeasible given the nature of the interventions; whilst the trial statistician was blind to allocations.

Interventions

Men were randomised to both a physical activity intervention (two levels; brisk walking v control) and a nutrition intervention (three levels; modified nutrition group v lycopene v control). Men in the physical activity intervention group were instructed to walk at a brisk pace for 30 minutes on at least 5 days a week in addition to their usual physical activity, whilst control group men were asked to continue with their usual physical activity. Men in the plant-based diet group (modified nutrition) were instructed to eat as many portions of fruit and vegetables as possible a day, aiming for at least 5 daily portions (printed instructions explained how to measure a portion). In addition, men in the plant-based diet group were asked to reduce their dairy milk intake as much as possible and to use a non-dairy alternative, for example soy, almond or rice milk as often as possible. Men in the lycopene group were asked to consume one 10mg lycopene capsule daily (Holland and Barrett, a UK supplier

For peer review only - http://bmjopen.bmj.com/site/about/guidelines.xhtml

For peer review only

of nutritional supplements, supplied to the men and paid for by the trial). Men in the nutrition control group were asked to continue with their usual diet. Control men who asked for physical activity or nutrition advice were provided with publicly available information. Instructions on how to perform the interventions were administered by the research nurse. All men were asked to follow their allocated interventions for six months and were contacted at one, two, five, eight, 13, 15 and 18 weeks post-randomisation. The method of contact was chosen by the man: text message, emails, phone call or post. These communications contained recipe ideas (plant based diet group) and theory developed motivational messages to encourage continuation of the trial-group intervention [(16)].

Data Collection

Men attended the hospital for a research nurse appointment at trial baseline (randomisation), three and six months and completed patient-reported outcome measures (PROMs) (paper questionnaires). If the man was unable to attend the hospital, the questionnaire was sent in the post. Questionnaires analysed here to illustrate the impact of adherence were the Recent Physical Activity Questionnaire (RPAQ) [(17)] and Food Frequency Questionnaire (FFQ) [(18)] whilst the other PROMs are detailed elsewhere [(16)]. At baseline, men reported socio-demographic information (level of education, marital status, occupation and ethnicity), and family medical history of cancer. At baseline, three and six months, smoking and alcohol use, and nurse-measured anthropometry, were recorded. Non-fasting blood samples were collected at baseline and six months. All men completed a paper daily monitoring form for the six-month duration logging daily step count, additionally; the physical activity intervention group recorded the duration of brisk walking in minutes; the plant-based diet nutrition group recorded the daily number of portions of fruit or vegetables consumed and the amount of dairy milk replaced by non-dairy alternatives (all / some / none); whilst the lycopene supplementation group recorded whether they had taken the lycopene supplement (yes / no).

For peer review only - http://bmjopen.bmj.com/site/about/guidelines.xhtml

Public and Patient Involvement (PPI)

A prostate cancer PPI group was involved from the early protocol development stages. The PPI group contributed to discussions regarding intervention content and length, reviewed trial documentations, such as consent forms and participant information sheets, revised intervention instructions to improve readability and completed questionnaires to assess participant burden.

Sample Size Calculation

The sample size calculation, described previously [16], identified a required minimum sample size of 75 men to estimate an adherence rate (the primary outcome, see below) of 75% with a confidence interval of ± 5 % (90% power, p=0.05) in each group. We aimed to recruit at least 100 men to the cohort study, anticipating an 80% randomisation rate.

Statistical Analysis

Analyses included all participants with measurements for the primary outcomes and trial baseline values at randomisation in an intention to treat analysis. All statistical analyses used Stata 14 [19].

Primary outcomes

The primary outcomes were: i) the randomisation rate (the number of men agreeing to be randomised into the RCT divided by the total number of men in the cohort) and ii) adherence to the man's allocated intervention at six months following randomisation. Adherence was based on the four weeks prior to the participants' latest entry in their daily monitoring form. Specifically, for each intervention; to assess adherence to the plant-based diet we analysed the proportion of days that the men reported they consumed five or more portions of fruit or vegetables, and the proportion of days they replaced all or some of their dairy milk with non-dairy alternative. Adherence to the lycopene intervention was based on the proportion of days that the men reported they took the

For peer review only

lycopene supplement. The threshold for acceptable adherence was 90%, which equates to adhering to the fruit and vegetable or lycopene interventions on 26 out of a possible 28 days. Adherence to the physical activity intervention was based on number of days of doing 30 or more minutes of self-reported brisk walking. Across the 28 days over which adherence was monitored, men were considered to have adhered 100% to the physical activity intervention if they did 30+ minutes of walking on at least 20 days, while 90% adherence was defined as doing 30+ minutes of walking on 18 of the 28 days.

Secondary outcomes

PrEvENT secondary outcomes were trial retention, blood biomarkers of fruit and vegetable consumption, self-reported food consumption (measured using the FFQ), self-reported physical activity (measured using the RPAQ), trial tolerability (measured by qualitative interviews and adverse events); these outcomes are reported here. Other secondary outcomes were change in prostate specific antigen and insulin-like growth factor, change in urinary symptoms, psychological factors, health beliefs, quality of life, fatigue, general health, accelerometer and pedometer data and lifestyle behaviours (smoking and drinking). These latter secondary outcomes are not reported here.

As secondary measures of adherence in the dietary interventions serum concentrations of lutein, zeaxanthin, β-cryptoxanthin, α-carotene, β-carotene and lycopene were measured by reverse-phase high-performance liquid chromatography (HPLC) [20]. Assays were standardised against appropriate National Institute of Standards and Technology reference materials [21]. These assays are also externally quality assured by participation in the French Society for Vitamins and Biofactors quality assurance scheme [22].

Mean portions of fruit and vegetables consumed per day were calculated from the FFQ. Physical activity energy expenditure was derived from the RPAQ. The effects of the nutrition and physical

For peer review only - http://bmjopen.bmj.com/site/about/guidelines.xhtml

activity interventions on fruit and vegetable consumption and energy expenditure, respectively, were assessed using multivariable linear regression. As pre-specified, we assumed no interaction between the interventions and tested this through repeating the multivariable linear regression analyses with an interaction term between the interventions, and application of the Wald test with the null hypothesis that the interaction terms were zero. We also compared the consumption of non-dairy milk recorded in the FFQ (categorised into 'Dairy': full cream, semi skimmed, skimmed or dried milk; and 'Non-dairy': soya, other) and blood biomarkers of fruit and vegetable consumption (Lutein, Zeaxanthin, Beta cryptoxanthin, Alpha carotene, Beta carotene, Lycopene) between trial groups using chi-square tests for categorical outcomes and Kruskal-Wallis tests for continuous outcomes respectively. A Bonferroni correction was applied to P values (multiplied by two) for post-hoc pairwise comparisons between the nutrition groups (no comparison was made between the lycopene and plant-based interventions) calculated using pairwise chi-square tests for categorical outcomes and Wilcoxon rank-sum tests for continuous outcomes. Intervention tolerability was assessed via adverse events and post-trial qualitative interviews (reported elsewhere).

Exploratory outcomes

In order to describe change in adherence from the beginning to the end of the trial period adherence was also calculated for the first four weeks of daily data monitoring forms after the participants earliest entry.

RESULTS

Study Recruitment, Participant Characteristics, Randomisation and Follow-up

Between August 2014 and May 2016, 292 eligible men scheduled for radical prostatectomy were invited to participate and of these, 108 (37.0 %) entered the cohort study (Figure 1). The most common reasons for not enrolling were: distance to travel (n=75), not being contactable (n=58), or uninterested in taking part (n=28). Of the 108 men, 96 completed cohort follow up, a further 9 were

For peer review only - http://bmjopen.bmj.com/site/about/guidelines.xhtml

no longer eligible for the RCT, thus 87 were invited into the RCT and 81 (93.1%, 95% CI 85.8% to 96.8%) consented to be randomised, a trial primary endpoint.

INSERT FIGURE 1: CONSORT diagram

Men were randomised on average around 8 weeks after surgery (range: 4 weeks (31 days) to 15 weeks (108 days)). The men were on average 64.0 years of age (sd = 6.3 years), with 88% (n = 68) self-reporting as white British and with a median prostate specific antigen of 8.7 ng/ml (inter-quartile range IQR: 5.9 – 12.3). Almost all men were former or never smokers (97.4%, n = 75), and 20% (n = 16) self-reported as consuming more than 14 units of alcohol per week. In general, baseline characteristics were similarly distributed between trial groups (Table 1). Seventy-five of the 81 men completed the trial (92.6 %), three were lost to follow-up and three withdrew. Those who withdrew were for reasons unrelated to the interventions. We present the results of the 75 men who completed the trial.

INSERT TABLE 1: Participant characteristics, baseline measures and clinical data by intervention group

Primary Outcomes:

Adherence to interventions by self-reported daily records

Adherence for at least 90% of the time was 40.0% (95% CI 23.4% to 59.3%, n = 10 of 25) in the plant-based diet group for the fruit and vegetable component and 72.0% (95% CI 52.4% to 85.7%, n = 18 of 25) for the dairy milk component. In the lycopene group, 78.6% (95% CI 60.5% to 89.8%, n = 22 of 28) of men adhered at least 90% of the time. Adherence to the physical activity intervention was 53.8% (95% CI 38.6% to 68.4%, n = 21 of 39) at least 90% of the time (Table 2).

For peer review only - http://bmjopen.bmj.com/site/about/guidelines.xhtml

INSERT TABLE 2: Adherence to the interventions by self-reported daily monitoring

Secondary outcomes:

Effects of the interventions on self-reported diet and physical activity PROMs

Those men in the plant-based diet intervention group reported consuming a mean of 9.9 portions (sd = 4.7 portions) of fruit and vegetables a day based on the FFQ PROMs. This was almost four portions (adjusted difference = 3.7, 95% CI 1.1 to 6.4 portions/day) more than those in the control group (mean = 6.2, sd = 3.8 portions) (P=0.007). Those in the lycopene intervention groups reported consuming a mean of 8.2 portions per day (sd = 4.8 portions), which was 2.1 portions (95% CI -0.5 to 4.7) more per day than the control group (p=0.12) (Table 3). Consumption of non-dairy milk was highest in the plant-based diet intervention group (78.3%, 95% CI 58.1% to 90.3%, n=18 of 23), and was very low otherwise (7.7%, 95% CI 2.1% to 24.1%, 2 of 26; and 0%, 95% CI 0.0% to 16.1%, 0 of 20 for the lycopene intervention and control groups respectively, P<0.001) (Table 3).

INSERT TABLE 3: Effects of interventions on fruit and vegetable consumption, non-dairy milk consumption and energy expenditure at six months

Those men in the physical activity intervention and control groups reported very similar mean energy expenditure based on the PROMs data (mean = 39.7 kj/kg/d, sd = 26.3 kj/kg/d; and mean = 40.8 kj/kg/d, sd = 28.6 kj/kg/d, respectively) (adjusted difference = -0.8 kj/kg/d, 95% CI -13.6 to 11.9 kj/kg/d, p=0.90) (Table 3).

There was no evidence of an interaction between the nutrition and physical activity interventions (F=0.65, P=0.63) so the data are not shown here.

Effects of the nutrition intervention on blood biomarkers of fruit and vegetable consumption

For peer review only - http://bmjopen.bmj.com/site/about/guidelines.xhtml

Lutein (P = 0.07), beta cryptoxanthin (P = 0.07), beta carotene (P = 0.09) and lycopene (P = 0.07) were higher in the plant-based intervention group than the control group. There was no difference in lycopene between the lycopene intervention group compared with the control group (P=0.64). No other differences were found between the blood biomarkers (Table 4).

INSERT TABLE 4: Effects of interventions on blood biomarkers and non-dairy milk consumption at six months

Intervention tolerability

Nine adverse events, self-reported by participants, were classed as unrelated to the interventions and included fever, alcohol withdrawal symptoms, urinary symptoms, diverticulitis, pneumonia, *Campylobacter* infection and hernia repair procedure. Three adverse events were considered as "possibly related to the interventions", these were indigestion, abdominal bloating and knee pain. Data from qualitative interviews reported elsewhere also suggested that men found all the interventions to be acceptable, easy to accommodate and well tolerated [23].

Exploratory analysis

Change in adherence from the beginning to the end of the trial period was examined. In the plant-based group there was an increase in adherence at 6 months compared to the start of the intervention, both for the fruit and vegetable consumption which increased from 20.0% (95% CI 8.9% to 39.1%, n =5 of 25) and the reduced dairy consumption, which increased from 60.0% (95% CI 40.7% to 76.6%, n = 15). Adherence in the lycopene group fell slightly from 89.3% (95% CI 72.8% to 96.3%, n = 25 of 28) within the first four weeks and in the physical activity group which fell slightly from 59.0% (95% CI 43.4% to 72.9%, n = 23 of 39) during the first four weeks to reported adherence at 6-month follow up (Table 5).

For peer review only - http://bmjopen.bmj.com/site/about/guidelines.xhtml

INSERT TABLE 5: Exploratory data - adherence to the interventions by self-reported daily monitoring (first four weeks)

DISCUSSION

PrEvENT recruited 37.0% of eligible men into the cohort and randomised 93.1% of men from the cohort into the RCT. This is higher than our anticipated randomisation rate of 80%. Forty percent of men in the plant-based diet group adhered to the fruit and vegetable component of the intervention at six months, with more than 70% adhering to the dairy replacement component. Almost 80% of men in the lycopene group adhered to the intervention at six months and just under 54% of men adhered to the physical activity intervention at six months. Those in the plant-based diet consumed 9.9 portions of fruit and vegetables per day, which was more than the nutrition control (6.4 portions); as did the lycopene group, at 8.2 portions per day. Over 90% completed 6-month follow up.

Adherence for both elements of the plant-based intervention increased from the first month of the intervention to the last month. This may reflect the men's need to develop new shopping, eating and social behaviours to incorporate the increased fruit and vegetable consumption as well as the reduction in dairy milk. It should be noted that our definition of adherence was strict, expecting participants to follow the intervention more than 90% of the time. Previous diet and physical activity interventions in cancer populations have reported higher adherence rates of around 70%. However, in these instances the interventions were either less intensive or definitions of adherence were not as strict as the current study [24-26].

The findings from our blood biomarker analysis further contribute to the conclusion of acceptable adherence, with higher levels of lutein and beta cryptoxanthin evident in the plant-based population at six months, compared to the other groups. Both carotenoids are found in fruit and vegetables, for

For peer review only - http://bmjopen.bmj.com/site/about/guidelines.xhtml

example dark leafy greens, oranges, red peppers and carrots, thus implying higher consumption of certain fruits and vegetables in the plant-based diet group compared to the other groups. Almost 80% of the plant-based group self-reported consuming non-dairy milk products at six months compared to 8% of the lycopene group and none of the control group, suggestive of high compliance with that intervention.

Adherence to lycopene supplements was high at almost 80% at six months, a slight drop from the first four weeks of the trial where almost 90% adherence was reported. This may have been down to the simplicity of taking a lycopene supplement, where the majority of men were able to easily incorporate the capsule into their daily routine [23]. It should be noted that despite excellent adherence to the lycopene supplementation, no differences in lycopene levels were reported in the blood biomarkers between men in the lycopene and other groups. Assuming self-reported lycopene supplementation consumption was correct, this finding may suggest the supplement dosage of 10mg per day was not high enough. This should be considered when designing future lycopene interventions. Our high adherence rates, however, confirm the feasibility of this population taking a daily lycopene supplement, which was an outcome of the trial.

The plant-based adherence rates were lower than the anticipated 75%, although adherence to the reduced dairy milk component were higher, almost reaching this target; the lycopene adherence rates were higher than the proposed 75%. Almost 54% of participants adhered to the physical activity intervention at six months, slightly lower than the 59% adhering within the first four weeks of the trial.

Overall, these randomisation and adherence rates suggest that men were willing to take part and generally followed the intervention instructions well.

For peer review only

For peer review only - http://bmjopen.bmj.com/site/about/guidelines.xhtml

The finding that participants in the plant-based diet intervention group consumed four additional daily portions of fruit or vegetables than the control group is substantial from a public health perspective. The lycopene intervention group also consumed a significantly larger amount of fruit and vegetables than the control group, although less than the plant-based diet participants. This may have been for one of two reasons. Firstly, the information sheet explained that lycopene is found in red food products such as tomatoes and grapefruit, thus the lycopene group may have increased fruit and vegetable consumption for this reason, as well as taking the supplement. Secondly, it may have been the case that being in an active nutrition group, regardless of the specific instructions, increased participants awareness about their nutrition, which may have resulted in them consuming more fruit and vegetables [11].

Overall consumption of fruit and vegetables of all participants was reported to be high. Seven or eight portions per day were reported at baseline, increasing to eight to ten portions at six months (nutrition control was slightly lower at six portions). This is proportionately higher than the national average. In 2013/2014, 27% of those aged 19 to 64 years and 35% of those aged 65 years and over met the 5-A-Day recommendation [27, 28]. The average consumption for these age groups were 4 and 4.2 portions of fruit and vegetables per day, respectively [28]. There may be a number of explanations for this. Due to the information in the public domain surrounding prostate cancer, which includes general advice to eat a healthy balanced diet, many of the men may have increased their fruit and vegetable consumption at time of diagnosis [10, 11]. Alternatively, as all participants had already participated in the cohort, which asked questions about their diet, this may have made them consider their diet prior to entering the RCT and thus increased consumption [11]. Finally, there may have been some over-reporting of fruit and vegetable consumption, either due to social desirability, inconsistent recall or as a result of previously reported issues with the FFQ [29].

For peer review only - http://bmjopen.bmj.com/site/about/guidelines.xhtml

There were no differences in levels of energy expenditure reported between the physical activity intervention group and control group. It may have been the case that no differences existed; or that the RPAQ was not sensitive enough to detect differences. Alternatively, it may have been that the physical activity intervention was not vigorous enough to warrant a difference in score between the physical activity intervention groups. This is something to be noted for future physical activity interventions, with the possibility of increasing the intensity of the intervention.

Limitations:

There are some limitations. Except for the statistician, all researchers and participants were unblinded. Men were provided with information on the specifics of all trial groups and therefore there may have been cross over in behaviour, potentially diluting results. However, blinding was not feasible given the nature of the intervention. The population lacked heterogeneity, particularly with regards to ethnicity, possibly limiting the generalisation of results. Many of the key variables were assessed via PROMs, potentially causing responder bias. However, we also report objective biochemical measures which supported the inference of good adherence, particularly within the plant-based group. It should be noted that the two key reasons for not enrolling were having too far to travel to attend research clinics or no response to the invitation being received. If these two barriers were removed, then our data suggests that the cohort recruitment could potentially have increased to 51%.

Conclusion

The PrEvENT interventions were feasible as evidenced by high randomisation rates, high retention rates, excellent acceptability and tolerability. Men demonstrated good adherence to all interventions, particularly the lycopene supplement, brisk walking and the reduced dairy milk element of the plant-based diet interventions. Positive effects on fruit and vegetable consumption

For peer review only - http://bmjopen.bmj.com/site/about/guidelines.xhtml

were seen in the plant-based diet and lycopene groups. The development of a definitive RCT should consider: travel distance to research clinics; increasing the intensity of the physical activity intervention and dosage of the lycopene supplementation; and possibly reducing the minimum criteria for adherence.

ADDITIONAL INFORMATION

Ethics approval and consent to participate

The trial was performed in accordance with the Declaration of Helsinki, and the ethical principles of the International Conference on Harmonisation – Good Clinical Practice. All study procedures were approved by the National Research Ethics Committee South West – Cornwall and Plymouth (REC reference number 14/SW/0056). All men provided fully informed written consent at both the cohort and RCT recruitment stages. The trial was registered with the ISRCTN registry on 17th November 2014 (ISRCTN99048944).

Availability of data and materials

The datasets generated and analysed during the current study are available from the corresponding author on reasonable request.

Authorship

LHM conceived and designed the work, acquired and interpreted data and drafted the manuscript. CP interpreted data and drafted the manuscript. ES and LAR acquired data. CMP, JMPH, ER, AK, AB and RP designed the work. CS, LJ, KMB and AF acquired data. JVW and SG interpreted data. JO and PA designed the work. JAL and RMM conceived the work, designed the work and interpreted data. All authors revised the manuscript, approved the final version of the manuscript and agree to be accountable for all aspects of the work.

For peer review only

Acknowledgements

The authors wish to acknowledge Mr Stu Toms for his development of the trial database and data management support throughout.

Funding

This study was supported by the NIHR Biomedical Research Centre at University Hospitals Bristol NHS Foundation Trust and the University of Bristol. The views expressed in this publication are those of the authors and not necessarily those of the NHS, the National Institute for Health Research or the Department of Health.

The Bristol Randomised Trials Collaboration (BRTC) receives National Institute for Health Research CTU Support Funding. The BRTC is a UK Clinical Research Collaboration registered CTU and a member of the NCRI Cancer Clinical Trials Group.

The views expressed are those of the authors and not necessarily those of the NHS, the NIHR, BRTC or the Department of Health.

For peer review only

Conflict of interest

The authors declare no conflict of interest.

References

1. Cancer Research UK. Prostate cancer statistics 2016 [02.06.2016]. Available from: http://www.cancerresearchuk.org/health-professional/cancer-statistics/statistics-by-cancer-type/prostate-cancer#heading-Zero].
2. Hackshaw-McGeagh LE, Perry RE, Leach VA, Qandil S, Jeffreys M, Martin RM. A systematic review of dietary, nutritional and physical activity interventions for the prevention of prostate cancer progression and mortality. Cancer Causes Control. 2015;26:1521-50.
3. World Cancer Research Fund. Continuous Update Project Report: Diet, nutrition, physical activity and prostate cancer. 2014.

For peer review only - http://bmjopen.bmj.com/site/about/guidelines.xhtml

4. Chan JM, Holick CN, Leitzmann MF, Rimm EB, Willett WC, Stampfer MJ. Diet after diagnosis and the risk of prostate cancer progression, recurrence, and death (United States). Cancer Causes Control. 2006;17:199-208.
5. Neill MG, Fleshner NE. An update on chemoprevention strategies in prostate cancer for 2006. Curr Opin Urol. 2006;16:132-7.
6. Downer MK, Batista JL, Mucci LA, Stampfer MJ, Epstein MM, Hakansson N, Wolk A, Johansson JE, Andren O, Fall K, Andersson SO. Dairy intake in relation to prostate cancer survival. Int J Cancer. 2017;140(9):2060-9.
7. Kruk J, Aboul-Enein H. What Are the Links of Prostate Cancer with Physical Activity and Nutrition? : A Systematic Review Article. Iranian Journal of Public Health. 2016;45(12):1558-67.
8. Physical Activity Guidelines Advisory Committee. Physical Activity Guidelines Advisory Committee Report, 2008. 2008.
9. Studenski S PS, Patel K, Rosano C, Faulkner K, Inzitari M, Brach J, Chandler J, Cawthon P, Connor EB, Nevitt M, Visser M, Kritchevsky S, Badinelli S, Harris T, Newman AB, Cauley J, Ferrucci L, Guralnik J. Gait speed and survival in older adults. JAMA. 2011;305(1):50 - 8.
10. Hackshaw-McGeagh LE, Penfold CM, Walsh E, Donovan JL, Hamdy FC, Neal DE, Jeffreys M, Martin RM, Lane JA. Physical activity, alcohol consumption, BMI and smoking status before and after prostate cancer diagnosis in the ProtecT trial: opportunities for lifestyle modification. Int J Cancer. 2015;137(6):1509-15.
11. Avery KNL, Donovan JL, Gilbert R, Davis M, Emmett P, Down L, Oliver S, Neal DE, Hamdy FC, Lane JA. Men with prostate cancer make positive dietary changes following diagnosis and treatment. Cancer Causes & Control. 2013;24(6):1119-28.
12. Karlsen RV, Bidstrup PE, Christensen J, Larsen SB, Tjonneland A, Dalton SO, Johansen C. Men with cancer change their health behaviour: a prospective study from the Danish diet, cancer and health study. Br J Cancer. 2012;107(1):201-6.
13. Sutton E, Hackshaw-McGeagh LE, Aning J, Bahl A, Koupparis A, Persad R, Martin RM, Lane JA. The provision of dietary and physical activity advice for men diagnosed with prostate cancer: a qualitative study of the experiences and views of health care professionals, patients and partners. Cancer Causes & Control. 2017;28(4):319-29.
14. Demark-Wahnefried W, Aziz NM, Rowland JH, Pinto BM. Riding the crest of the teachable moment: promoting long-term health after the diagnosis of cancer. J Clin Oncol. 2005;23(24):5814-30.
15. Williams K, Steptoe A, Wardle J. Is a cancer diagnosis a trigger for health behaviour change? Findings from a prospective, population-based study. Br J Cancer. 2013;108(11):2407-12.
16. Hackshaw-McGeagh L, Lane JA, Persad R, Gillatt D, Holly JM, Koupparis A, Rowe E, Johnston L, Cloete J, Shiridzinomwa C, Abrams P, Penfold CM, Bahl A, Oxley J, Perks CM, Martin R. Prostate cancer - evidence of exercise and nutrition trial (PrEvENT): study protocol for a randomised controlled feasibility trial. Trials. 2016;17(1):123.
17. Besson H, Brage S, Jakes RW, Ekelund U, Wareham NJ. Estimating physical activity energy expenditure, sedentary time, and physical activity intensity by self-report in adults. Am J Clin Nutr. 2010;91(1):106-14.
18. Willett WC, Sampson L, Stampfer MJ, Rosner B, Bain C, Witschi J. Reproducibility and validity of a semiquantitative food frequency questionnaire. Am J Epidemiol. 1985;122.
19. StataCorp. Stata Statistical Software: Release 14. College Station, TX: StataCorp LP; 2015.
20. Craft N, Wise S, H. Soares J. Optimisation of an isocratic high performance liquid chromatography separation of carotenoids. Journal of Chromatography. 1992;589(1-2):171-6.
21. National Institute of Standards and Technology. Certification of Standard Reference Material® 968f Fat-Soluble Vitamins in Frozen Human Serum. 2017.
22. Societe Francaise Vitamines and Biofacteurs. 2011 Quality Assurance Program: Societe Francaise Vitamines and Biofacteurs; 2011 [15.02.2018]. Available from: http://www.sfvb.org/pages/aquality.htm.

For peer review only

23. Shingler E, Hackshaw-McGeagh L, Robles L, Persad R, Koupparis A, Rowe E, Shiridzinomwa C, Bahl A, Martin RM, Lane JA. The feasibility of the Prostate cancer: Evidence of Exercise and Nutrition Trial (PrEvENT) dietary and physical activity modifications: a qualitative study. Trials. 2017;18(1):106.

24. Koutoukidis DA, Beeken RJ, Manchanda R, Michalopoulou M, Burnell M, Knobf MT, Lanceley A. Recruitment, adherence, and retention of endometrial cancer survivors in a behavioural lifestyle programme: the Diet and Exercise in Uterine Cancer Survivors (DEUS) parallel randomised pilot trial. BMJ Open. 2017;7(10).

25. Demark-Wahnefried W, Clipp EC, Lipkus IM, Lobach D, Snyder DC, Sloane R, Peterson B, Macri JM, Rock CL, McBride CM, Kraus WE. Main Outcomes of the FRESH START Trial: A Sequentially Tailored, Diet and Exercise Mailed Print Intervention Among Breast and Prostate Cancer Survivors. Journal of Clinical Oncology. 2007;25(19):2709-18.

26. James EL, Stacey FG, Chapman K, Boyes AW, Burrows T, Girgis A, Asprey G, Bisquera A, Lubans DR. Impact of a nutrition and physical activity intervention (ENRICH: Exercise and Nutrition Routine Improving Cancer Health) on health behaviors of cancer survivors and carers: a pragmatic randomized controlled trial. BMC Cancer. 2015;15(1):710.

27. Department of Health. 5-A-Day [21/08/2017]. Available from: http://www.nhs.uk/Livewell/5ADAY/Pages/5ADAYhome.aspx.

28. Public Health England. National Diet and Nutrition Survey Results from Years 5 and 6 (combined) of the Rolling Programme (2012/2013 – 2013/2014). 2016.

29. Cade JE, Burley VJ, Warm DL, Thompson RL, Margetts BM. Food-frequency questionnaires: a review of their design, validation and utilisation. Nutrition Research Reviews. 2007;17(1):5-22.

For peer review only

For peer review only - http://bmjopen.bmj.com/site/about/guidelines.xhtml

Table 1: Participant characteristics, baseline measures and clinical data by intervention groups

	n (% of participants in group)				
	Nutrition (n=81)			Physical activity (n=81)	
	Lycopene intervention (n=28, 34.6%)	Plant based diet intervention (n=27, 33.3%)	Nutrition control (n=26, 32.1%)	PA control (n=39, 48.1%)	PA intervention (n=42, 51.9%)
**Participant characteristics**					
**Age (years)***	62.9 (7.7)	64.6 (5.7)	64.7 (5.5)	62.5 (6.9)	65.5 (5.5)
**Ethnicity**					
White British	23 (88.5)	23 (85.2)	22 (91.7)	33 (86.8)	35 (89.7)
**Highest education level^**					
Standard or less (e.g. o-levels, GCSE)	7 (26.9)	12 (44.4)	14 (60.9)	20 (52.6)	13 (34.2)
Further education (e.g. A-levels, HND, University degree)	19 (73.1)	15 (55.6)	9 (39.1)	18 (47.4)	25 (65.8)
**Marital status**					
Married / in a relationship	23 (88.5)	22 (81.5)	22 (95.7)	33 (86.8)	34 (89.5)
**Father had prostate cancer**					
Yes	11 (44.0)	2 (7.4)	1 (4.3)	8 (21.6)	6 (15.8)
**Smoking status**					
Current	0 (0.0)	0 (0.0)	2 (7.7)	1 (2.7)	1 (2.5)
Former	9 (37.5)	13 (48.1)	16 (61.5)	21 (56.8)	17 (42.5)
Never	15 (62.5)	14 (51.9)	8 (30.8)	15 (40.5)	22 (55.0)
**Alcohol consumption (units/week)[+]**	1.0 (0.0; 7.0)	4.0 (0.0; 12.0)	4.5 (0.0; 22.0)	2.0 (0.0; 7.0)	4.0 (0.0; 13.0)
**Alcohol consumption (categorised)[#]**					
Non-drinker (0 units/week)	14 (50.0)	9 (33.3)	8 (30.8)	17 (43.6)	14 (33.3)
≤14 units/week	11 (39.3)	14 (51.9)	9 (34.6)	15 (38.5)	19 (45.2)
>14 units/week	3 (10.7)	4 (14.8)	9 (34.6)	7 (17.9)	9 (21.4)
**Body mass index (kg/m^2)***	26.2 (3.6)	26.7 (3.6)	26.8 (3.1)	26.6 (3.5)	26.6 (3.3)
**Baseline measures and clinical data**					
**Pre-surgical Prostate specific antigen (ng/mL)[+]**	7.8 (5.2; 11.0)	8.4 (5.7; 9.6)	10.6 (8.4; 15.0)	8.4 (5.7; 11.0)	8.9 (6.4; 12.7)

For peer review only - http://bmjopen.bmj.com/site/about/guidelines.xhtml

**Baseline fruit and vegetable consumption (portions/day)***	7.5 (4.7)	8.5 (5.7)	7.3 (2.4)	8.2 (5.2)	7.4 (3.7)
**Baseline energy expenditure (kJ/kg/d)***	27.2 (32.9)	22.8 (13.9)	27.4 (21.6)	23.1 (23.0)	28.3 (25.0)
**Type of milk consumed most often**					
Dairy	22 (88.0)	22 (84.6)	24 (96.0)	30 (83.3)	38 (95.0)
Non-dairy	3 (12.0)	4 (15.4)	1 (4.0)	6 (16.7)	2 (5.0)
**Lutein - umol/l[+]**	0.36 (0.28; 0.55)	0.40 (0.32; 0.52)	0.35 (0.20; 0.46)	0.35 (0.28; 0.52)	0.38 (0.28; 0.51)
**Zeaxanthin - umol/l[+]**	0.11 (0.09; 0.16)	0.10 (0.09; 0.12)	0.10 (0.06; 0.13)	0.11 (0.08; 0.12)	0.10 (0.08; 0.14)
**Beta cryptoxanthin - umol/l[+]**	0.22 (0.16; 0.36)	0.25 (0.15; 0.36)	0.19 (0.11; 0.39)	0.22 (0.14; 0.32)	0.24 (0.14; 0.40)
**Alpha carotene - umol/l[+]**	0.07 (0.05; 0.09)	0.08 (0.06; 0.10)	0.07 (0.05; 0.08)	0.07 (0.05; 0.08)	0.07 (0.06; 0.09)
**Beta carotene - umol/l[+]**	0.64 (0.46; 1.06)	0.72 (0.50; 1.16)	0.56 (0.47; 0.84)	0.66 (0.47; 0.89)	0.64 (0.48; 1.07)
**Lycopene - umol/l[+]**	1.35 (1.03; 1.69)	1.24 (0.92; 1.44)	1.20 (0.90; 1.59)	1.23 (0.92; 1.47)	1.29 (1.04; 1.62)

* Values are mean (s.d) [+] Values are median (25%; 75%) ^ O-level, GCSE: national school examinations at age 16 years. A-level: national school examinations at age 18 years. HND (Higher National Diploma) is a work-related course provided by higher and further education colleges in the UK. A full-time HND takes two years to complete, and generally is the equivalent to two years at university # Non-drinker: 0 units/week, Moderate: >0 & ≤14 units/week, Hazardous: >14 & ≤50 units/week, Harmful: >50 units/week

Table 2: Adherence to the interventions by self-reported daily monitoring[1]

[1] Adherence was based on the four weeks prior to the participants' latest entry in their daily monitoring form. The threshold for acceptable adherence was 90%, which equates to adhering to the fruit and vegetable or lycopene interventions on 26 out of a possible 28 days. Adherence to the physical activity intervention equates to doing 30+ minutes of walking on 18 of the 28 days.

For peer review only

For peer review only - http://bmjopen.bmj.com/site/about/guidelines.xhtml

	Adherence to lycopene intervention[1]	Adherence to plant based diet intervention[1]		Adherence to physical activity intervention[1]
		Fruit and vegetables	Dairy replacement	
**Physical activity (PA)**				
PA control (N=36)	30.6% (n = 11)	13.9% (n = 5)	30.6% (n = 11)	-
PA intervention (N=39)	28.2% (n = 11)	12.8% (n = 5)	18.0% (n = 7)	53.8% (n = 21)
**Nutrition**				
Lycopene intervention (N=28)	78.6% (n = 22)	-	-	28.6% (n = 8)
Plant based diet intervention (N=25)	-	40.0% (n = 10)	72.0% (n = 18)	28.0% (n = 7)
Nutrition control (N=22)	-	-	-	27.3% (n = 6)

For peer review only

576 **Table 3: Effects of interventions on fruit and vegetable consumption, non-dairy milk consumption and energy expenditure at six months**

	Nutrition (n=75)			Physical Activity (n=75)	
	Lycopene intervention (n=28)	Plant based diet intervention (n=25)	Nutrition control (n=22)	Physical activity control (n=36)	Physical activity intervention (n=39)
Fruit and veg (portions/day), mean (sd)	8.2 (4.8)	9.9 (4.7)	6.2 (3.8)	8.3 (4.9)	8.1 (4.5)
Adjusted difference in fruit and vegetable consumption (portions/day) compared to the control group* (95% CI)	2.07 (-0.51-4.65)	3.73 (1.07-6.38)	-	-	-0.20 (-2.37 – 1.98)
P value	0.12[1]	0.007[1]			0.86[1]
Energy expenditure (kJ/kg/d), mean (sd)	41.8 (23.7)	43.1 (32.0)	34.9 (26.1)	40.8 (28.6)	39.7 (26.3)
Adjusted difference in energy expenditure (kJ/kg/d) compared to the control group* (95% CI)	6.91 (-8.77 – 22.6)	8.19 (-7.89 – 24.3)	-	-	-0.84 (-13.6 – 11.9)
P value	0.38[1]	0.31[1]			0.90[1]
**Type of milk consumed most often**					
Dairy, n (%)	24 (92.3)	5 (21.7)	20 (100.0)	22 (66.7)	27 (75.0)
Non-dairy, n (%)	2 (7.7)	18 (78.3)	0 (0.0)	11 (33.3)	9 (25.0)
P-values	*0.41[3]*	*<0.001[3]*	<0.001[2]		0.08[2]

577 ***** **-** fruit and vegetable consumption adjusted for physical activity intervention group, and energy expenditure adjusted for nutrition intervention group

For peer review only - http://bmjopen.bmj.com/site/about/guidelines.xhtml

[1] P-values from multivariate linear regression, [2] P-values from Wilcoxon sign-rank tests, [3] P-values from Wilcoxon sign-rank test with Bonferroni correction

For peer review only

Table 4: Effects of interventions on blood biomarkers and non-dairy milk consumption at six months

	**Nutrition (n=75)**						**Physical Activity (n=75)**		
	**Lycopene intervention (n = 28) Median (IQR)**	**Plant based diet intervention (n = 25) Median (IQR)**	**Nutrition control (n = 22) Median (IQR)**	**P** [1]	**P** (pairwise comparisons) [2]		**Physical Activity intervention (n = 39) Median (IQR)**	**Physical Activity control (n = 36) Median (IQR)**	**P**[1]
					**Lycopene vs control**	**Plant based vs control**			
**Lycopene** (umol/l)	1.30 (0.96; 1.60)	1.41 (1.12; 1.79)	1.16 (0.83; 1.39)	0.11	0.64	0.07	1.27 (0.95; 1.71)	1.36 (0.97; 1.58)	0.90
**Lutein** (umol/l)	0.37 (0.28; 0.46)	0.49 (0.36; 0.57)	0.36 (0.27; 0.48)	0.05	1.00	0.07	0.40 (0.29; 0.49)	0.38 (0.26; 0.55)	0.56
**Zeaxanthin** (umol/l)	0.09 (0.07; 0.16)	0.12 (0.10; 0.16)	0.10 (0.08; 0.14)	0.26	0.92	0.22	0.12 (0.08; 0.15)	0.11 (0.08; 0.16)	0.89
**Beta cryptoxanthin** (umol/l)	0.22 (0.11; 0.39)	0.30 (0.23; 0.51)	0.23 (0.12; 0.29)	0.06	1.00	0.07	0.24 (0.13; 0.50)	0.23 (0.16; 0.35)	0.44
**Alpha carotene** (umol/l)	0.07 (0.06; 0.09)	0.08 (0.06; 0.13)	0.07 (0.05; 0.09)	0.26	1.00	0.28	0.07 (0.05; 0.10)	0.07 (0.06; 0.09)	0.52
**Beta carotene** (umol/l)	0.59 (0.37; 0.97)	0.81 (0.54; 1.24)	0.57 (0.46; 0.79)	0.11	1.00	0.09	0.73 (0.45; 1.16)	0.64 (0.43; 0.93)	0.47

[1] P-values from Kruskal-Wallis tests unless stated otherwise, [2] P-values from Wilcoxon rank-sum tests, with Bonferroni correction unless stated otherwise

umol/l – micromole per litre.

For peer review only - http://bmjopen.bmj.com/site/about/guidelines.xhtml

583 **Table 5: Exploratory data - adherence to the interventions by self-reported daily monitoring (first four weeks)[2]**

	Adherence to lycopene intervention[2]	Adherence to plant based diet intervention[2]		Adherence to physical activity intervention[2]
		Fruit and vegetables	Dairy replacement	
**Physical activity**				
PA control (N=36)	**33.3% (n = 12)**	**2.8% (n = 1)**	**19.4% (n = 7)**	-
PA intervention (N=39)	**33.3% (n = 13)**	**10.3% (n = 4)**	**20.5% (n = 8)**	**59.0% (n = 23)**
**Nutrition**				
Lycopene intervention (N=28)	**89.3% (n = 25)**	-	-	**39.3% (n = 11)**
Plant based diet intervention (N=25)	-	**20.0% (n = 5)**	**60.0% (n = 15)**	**20.0% (n = 5)**
Nutrition control (N=22)	-	-	-	**31.8% (n = 7)**

[2] Adherence was based on the first four weeks of daily data monitoring forms after the participants earliest entry. The threshold for acceptable adherence was 90%, which equates to adhering to the fruit and vegetable or lycopene interventions on 26 out of a possible 28 days. Adherence to the physical activity intervention equates to doing 30+ minutes of walking on 18 of the 28 days.

For peer review only - http://bmjopen.bmj.com/site/about/guidelines.xhtml

For peer review only

586

For peer review only

For peer review only - http://bmjopen.bmj.com/site/about/guidelines.xhtml

Figure 1: CONSORT diagram

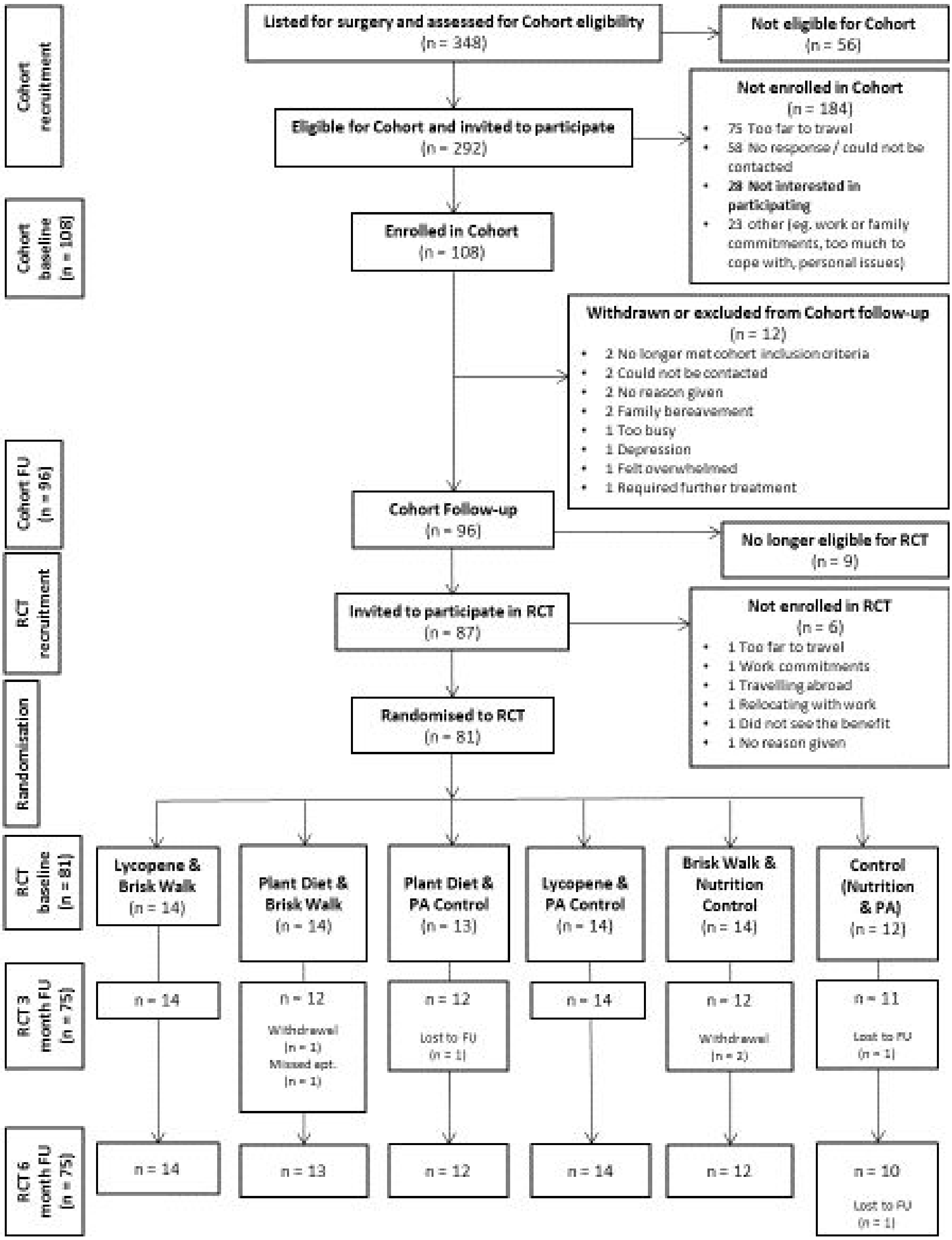

CONSORT 2010 checklist of information to include when reporting a randomised trial*

Section/Topic	Item No	Checklist item	Reported on page No
**Title and abstract**			
	1a	Identification as a randomised trial in the title	1
	1b	Structured summary of trial design, methods, results, and conclusions (for specific guidance see CONSORT for abstracts)	3-4
**Introduction**			
Background and objectives	2a	Scientific background and explanation of rationale	5-6
	2b	Specific objectives or hypotheses	6
**Methods**			
Trial design	3a	Description of trial design (such as parallel, factorial) including allocation ratio	6
	3b	Important changes to methods after trial commencement (such as eligibility criteria), with reasons	Na
Participants	4a	Eligibility criteria for participants	6
	4b	Settings and locations where the data were collected	6-7
Interventions	5	The interventions for each group with sufficient details to allow replication, including how and when they were actually administered	7
Outcomes	6a	Completely defined pre-specified primary and secondary outcome measures, including how and when they were assessed	9-11
	6b	Any changes to trial outcomes after the trial commenced, with reasons	Na
Sample size	7a	How sample size was determined	9
	7b	When applicable, explanation of any interim analyses and stopping guidelines	Na
Randomisation:			
Sequence generation	8a	Method used to generate the random allocation sequence	7
	8b	Type of randomisation; details of any restriction (such as blocking and block size)	7
Allocation concealment mechanism	9	Mechanism used to implement the random allocation sequence (such as sequentially numbered containers), describing any steps taken to conceal the sequence until interventions were assigned	7
Implementation	10	Who generated the random allocation sequence, who enrolled participants, and who assigned participants to interventions	7
Blinding	11a	If done, who was blinded after assignment to interventions (for example, participants, care providers, those	7

For peer review only - http://bmjopen.bmj.com/site/about/guidelines.xhtml

		assessing outcomes) and how	
	11b	If relevant, description of the similarity of interventions	7-8
Statistical methods	12a	Statistical methods used to compare groups for primary and secondary outcomes	9-11
	12b	Methods for additional analyses, such as subgroup analyses and adjusted analyses	11
**Results**			
Participant flow (a diagram is strongly recommended)	13a	For each group, the numbers of participants who were randomly assigned, received intended treatment, and were analysed for the primary outcome	CONSORT diagram (11-12)
	13b	For each group, losses and exclusions after randomisation, together with reasons	12
Recruitment	14a	Dates defining the periods of recruitment and follow-up	11
	14b	Why the trial ended or was stopped	Na
Baseline data	15	A table showing baseline demographic and clinical characteristics for each group	Table 1
Numbers analysed	16	For each group, number of participants (denominator) included in each analysis and whether the analysis was by original assigned groups	11-14
Outcomes and estimation	17a	For each primary and secondary outcome, results for each group, and the estimated effect size and its precision (such as 95% confidence interval)	11-14
	17b	For binary outcomes, presentation of both absolute and relative effect sizes is recommended	11-14
Ancillary analyses	18	Results of any other analyses performed, including subgroup analyses and adjusted analyses, distinguishing pre-specified from exploratory	11-14
Harms	19	All important harms or unintended effects in each group (for specific guidance see CONSORT for harms)	14
**Discussion**			
Limitations	20	Trial limitations, addressing sources of potential bias, imprecision, and, if relevant, multiplicity of analyses	18
Generalisability	21	Generalisability (external validity, applicability) of the trial findings	18
Interpretation	22	Interpretation consistent with results, balancing benefits and harms, and considering other relevant evidence	15-18
**Other information**			
Registration	23	Registration number and name of trial registry	4, 19
Protocol	24	Where the full trial protocol can be accessed, if available	20
Funding	25	Sources of funding and other support (such as supply of drugs), role of funders	20

*We strongly recommend reading this statement in conjunction with the CONSORT 2010 Explanation and Elaboration for important clarifications on all the items. If relevant, we also recommend reading CONSORT extensions for cluster randomised trials, non-inferiority and equivalence trials, non-pharmacological treatments, herbal interventions, and pragmatic trials. Additional extensions are forthcoming: for those and for up to date references relevant to this checklist, see www.consort-statement.org.

For peer review only - http://bmjopen.bmj.com/site/about/guidelines.xhtml

For peer review only

PHASE II RANDOMISED CONTROL FEASIBILITY TRIAL OF A NUTRITION AND PHYSICAL ACTIVITY INTERVENTION AFTER RADICAL PROSTATECTOMY FOR PROSTATE CANCER

Journal:	*BMJ Open*
Manuscript ID	bmjopen-2019-029480.R1
Article Type:	Research
Date Submitted by the Author:	17-Jul-2019
Complete List of Authors:	Hackshaw-McGeagh, Lucy E.; University of Bristol, NIHR Biomedical Research Centre<br>Penfold, Chris; University of Bristol, Musculoskeletal Research Unit<br>Shingler, Ellie; University of Bristol, NIHR Biomedical Research Centre<br>Robles, Luke; University of Bristol, Bristol Medical School: Population Health Sciences; University of Bristol, MRC Integrative Epidemiology Unit<br>Perks, Claire; University of Bristol, Bristol Medical School: Translational Health Sciences<br>Holly, Jeff; University of Bristol, Translational Health Sciences<br>Rowe, Edward; North Bristol NHS Trust, Urology<br>Koupparis, Anthony; North Bristol NHS Trust, Urology<br>Bahl, Amit; University Hospitals Bristol, Bristol Haematology and Oncology Centre<br>Persad, Raj; North Bristol NHS Trust<br>Shiridzinomwa, Constance; North Bristol NHS Trust, Clinical Research Centre<br>Johnson, Lyndsey; North Bristol NHS Trust, Clinical Research Centre<br>Biernacka, Kalina; University of Bristol, Translational Health Sciences<br>Frankow, Aleksandra; University of Bristol, Translational Health Sciences<br>Woodside, Jayne; Queens University Belfast, Institute for Global Food Security<br>Gilchrist, Sarah; Queens University Belfast, Institute for Global Food Security<br>Oxley, Jon; North Bristol NHS Trust, Department of Cellular Pathology<br>Abrams, Paul; Bristol Urological Institute, Southmead Hospital<br>Lane, Athene; University of Bristol, School of Social and Community Medicine; University of Bristol, NIHR Biomedical Research Centre<br>Martin, Richard; University of Bristol, Social Medicine; University of Bristol, NIHR Biomedical Research Centre
**Primary Subject Heading**:	Urology
Secondary Subject Heading:	Surgery, Research methods, Nutrition and metabolism
Keywords:	Prostate disease < UROLOGY, UROLOGY, Clinical trials < THERAPEUTICS, SURGERY, PREVENTIVE MEDICINE, Urological tumours < ONCOLOGY

SCHOLARONE™
Manuscripts

PHASE II RANDOMISED CONTROL FEASIBILITY TRIAL OF A NUTRITION AND PHYSICAL ACTIVITY INTERVENTION AFTER RADICAL PROSTATECTOMY FOR PROSTATE CANCER

L Hackshaw-McGeagh[1], C Penfold[2], E Shingler[1*], L A Robles[3,4*], C M. Perks[5], J M. P. Holly[5], E Rowe[6], A Koupparis[6], A Bahl[6], R Persad[6], C Shiridzinomwa[7], L Johnson[7], K M Biernacka[5], A Frankow[5], J V Woodside[8], S Gilchrist[8], J Oxley[9], P Abrams[6], J. A Lane[1,10,11*], R M Martin[1,4,10*]

Affiliation of author

[1] National Institute for Health Research (NIHR) Bristol Biomedical Research Centre (Nutrition Theme), Level 3, University Hospitals Bristol Education Centre, Upper Maudlin Street, Bristol, BS2 8AE

[2] National Institute for Health Research (NIHR) Bristol Biomedical Research Centre (Surgical Innovation Theme), Translational Health Sciences, Musculoskeletal Research Unit, University of Bristol, Level 2 Learning and Research Building, Southmead Hospital, Bristol, BS10 5NB

[3] Bristol Medical School: Population Health Sciences, University of Bristol, Oakfield House, Oakfield Grove, Clifton BS8 2BN

[4] MRC Integrative Epidemiology Unit, University of Bristol, Canynge Hall, 39 Whatley Road, Bristol, BS8 2PS

[5] Bristol Medical School: Translational Health Sciences, IGFs and Metabolic Endocrinology Group, University of Bristol, Level 2 Learning & Research Building, Southmead Hospital, Bristol, BS10 5NB

[6] Bristol Urological Institute, Level 3 Learning and Research Building, Southmead Hospital, Bristol, BS10 5NB

[7] Clinical Research Centre, North Bristol NHS Trust, Southmead Hospital, BS10 5NB

[8] Nutrition Group, Institute for Global Food Security (Centre for Public Health), School of Medicine, Dentistry and Biomedical Sciences, Queen's University Belfast, Institute of Clinical Science B (First Floor), Grosvenor Road, Belfast, BT12 6BJ

[9] Department of Cellular Pathology, Pathology Sciences Building, North Bristol NHS Trust, Southmead Hospital, Bristol, BS10 5NB

For peer review only

For peer review only - http://bmjopen.bmj.com/site/about/guidelines.xhtml

[10] Bristol Medical School: Population Health Sciences, University of Bristol, Canynge Hall, 39 Whatley Road, Bristol, BS8 2PS

[11] Bristol Randomised Trials Collaboration (BRTC), Bristol Medical School: Population Health Sciences, University of Bristol, Canynge Hall, 39 Whatley Road, Bristol, BS8 2PS

* These authors contributed equally to this work

Corresponding author

Dr JA Lane, Bristol Medical School: Population Health Sciences, University of Bristol, Canynge Hall, 39 Whatley Road, Bristol, BS8 2PS, 0117 928 7335, Athene.Lane@bristol.ac.uk

Word count: 4177

Tables and figures: 6

For peer review only

Abstract

Objective: Dietary factors and physical activity may alter prostate cancer progression. We explored the feasibility of lifestyle interventions following radical prostatectomy for localised prostate cancer.

Design: Patients were recruited into a pre-surgical observational cohort; following radical prostatectomy, they were offered randomisation into a 2x3 factorial randomised controlled trial (RCT).

Setting: A single National Health Service trust in the South West of England, UK.

Participants: Those with localised prostate cancer and listed for radical prostatectomy were invited to participate.

Randomisation: Random allocation was performed by the Bristol Randomised Trial Collaboration via an online system.

Interventions: Men were randomised into both a modified nutrition group (either increased vegetable and fruit, and reduced dairy milk; or lycopene supplementation; or control) and a physical activity group (brisk walking or control), for six months.

Blinding: Only the trial statistician was blind to allocations.

Primary outcome measures: Collected at trial baseline, three and six months, with daily adherence reported throughout. Primary outcomes were measures of feasibility: randomisation rates and intervention adherence at six months.

Results: 108 men entered the pre-surgical cohort and 81 were randomised into the post-surgical RCT (randomisation rate: 93.1%), 75 completed the trial. Of 25 men in the nutrition intervention, 10 (40.0%; 95% CI: 23.4%-59.3%) adhered to the fruit and vegetable recommendations and 18 (72.0% 95% CI 52.4%-85.7%) to reduced dairy intake. Adherence to lycopene (n=28), was 78.6% (95% CI: 60.5%-89.8%), whilst 21/39 adhered to the walking intervention (53.8%; 95% CI: 38.6%-68.4%). Most men were followed up at six months (75/81; 92.6%)**.** Three "possibly related" adverse events were indigestion, abdominal bloating and knee pain.

For peer review only - http://bmjopen.bmj.com/site/about/guidelines.xhtml

Conclusions: Interventions were deemed feasible, with high randomisation rates and good adherence. A definitive RCT is proposed.

Trial registration: ISRCTN 99048944

Funding: NIHR Biomedical Research Centre at University Hospitals Bristol NHS Foundation Trust and the University of Bristol.

Strengths And Limitations Of This Study

- Robust, gold standard, randomised control trial 2x3 design, with 6 month follow up
- Excellent retention rates, with only 6 participants withdrawing or being lost to follow up
- Outcomes include objective biochemical measures which are not open to responder bias
- Except for the statistician, all researchers and participants were unblinded; blinding was not feasible given the nature of the intervention.

For peer review only

INTRODUCTION

Prostate cancer is the most common male cancer in the UK. There were 47 740 new cases diagnosed in 2016 (1), accounting for 26% of UK male cancers. Furthermore, in 2016 there were 11 600 prostate cancer deaths, making the disease the second commonest cause of UK cancer-related death (1). Following improvements in clinical diagnosis and treatment leading to rising survival rates, more men are living longer with the disease and there is growing interest in developing lifestyle modifications for tertiary prevention of prostate cancer morbidity and mortality (2).

Evidence summarised by the World Cancer Research Fund (3) suggests that vegetables and fruit, and associated micronutrients (e.g. carotenoids, some vitamins, and flavonoids), may reduce prostate cancer risk, whilst calcium and dairy intake may increase risk. Our recent review of 44 randomised controlled trials (RCTs) of dietary, nutrition and physical activity interventions suggest various dietary interventions (e.g. low fat diet, soya and soya foods, selenium, lycopene or green tea) may reduce prostate cancer progression and mortality (2). However, many studies were assessed as being of low quality (with high or unclear risk of bias), underpowered, inadequately reported, of short duration or measuring surrogate outcomes, such as prostate specific antigen doubling time which has an uncertain relationship with prostate cancer progression. We therefore wanted to conduct a high quality, low risk of bias, adequately powered, well reported, six month trial to explore the feasibility of a simple, easy to implement 'real life', dietary and physical activity intervention.

Lycopene, an antioxidant phytonutrient, has been observed to reduce prostate cancer progression (4, 5), while high-fat milk intake has been positively associated with prostate cancer progression (6). However, robust RCT evidence for the effects of lycopene or milk intake in relation to prostate cancer progression is lacking (3).

For peer review only

Moderate-vigorous physical activity is another potentially promising intervention (7); evidence suggests that it is safe and can benefit quality of life throughout the cancer journey (8) but the evidence is also largely based on observational data: further RCTs are needed for definitive evidence. Moderate–vigorous intensity activity is defined as activities that cause sweating and increased heart and respiratory rate, of which brisk walking is an example (9). Brisk walking has been associated with anti-cancer cellular behaviour (10), and does not require specialised equipment or training.

Some men spontaneously and positively alter lifestyle behaviours on receiving a diagnosis of prostate cancer. However, for many men this is not the case (11-13) and additional interventions are necessary to promote and maintain behavioural change (14-16). Radical prostatectomy (surgical removal of the prostate) is a common treatment for localised disease (1). We established a 2 x 3 factorial RCT - the Prostate Cancer: Evidence of Exercise and Nutrition Trial (PrEvENT) - to explore the feasibility of introducing modified nutrition (increased vegetables and fruit combined with reduced dairy milk) or lycopene supplements and physical activity (brisk walking) interventions in men treated with radical prostatectomy for localised prostate cancer. Here we report the primary trial findings.

MATERIALS AND METHODS

Trial Design and Participants

The trial protocol has been published elsewhere (17). In brief, men from a single National Health Service (NHS) trust in the South West of England, UK, who were diagnosed with localised prostate cancer and listed for radical prostatectomy were invited to participate in PrEvENT between August 2014 and May 2016. There were two phases: (i) recruitment of men scheduled for radical prostatectomy into a baseline cohort for epidemiological characterisation (i.e. pre-surgical questionnaire data and blood sampling) of the target population; and ii) recruitment of the men once they had their radical prostatectomy into a 2x3 factorial RCT.

For peer review only - http://bmjopen.bmj.com/site/about/guidelines.xhtml

Cohort and RCT inclusion criteria were: localised prostate cancer; undergoing radical prostatectomy; capacity to provide informed consent; aged 18 or over; and sufficient understanding of the English language. Exclusion criteria were: inability to give informed consent; unavailability for follow-up; identified as unsuitable to participate by the treating clinician; co-morbidities (this could include uncontrolled congestive heart failure or angina, recent myocardial infarction or breathing difficulties requiring oxygen use or hospitalisation), allergies or religious beliefs which could prevent participation in the intervention (RCT only); regularly taking lycopene supplements or routinely exercising vigorously (RCT only).

Men were approached to participate in the cohort by their clinical care team, or a research nurse. Nurse-led consent at cohort recruitment included optional approval for prostate tissue extracted during surgery to be used for research purposes and willingness to be contacted about future involvement in further research. Approximately six weeks after surgery, the men who were participating in the cohort were invited to participate in the RCT. Six weeks post-surgery was identified as the ideal time to approach men to participate in the trial, as this is the time point where men are generally informed by their clinical team that they should be able to begin exercising again after radical prostatectomy.

Randomisation

Random allocation was performed by the Bristol Randomised Trial Collaboration (BRTC) via an online system to ensure that the recruiting nurses could not uncover allocation of trial group in advance (concealment of allocation). The research nurse, research team and men were not blind to intervention allocation because this would have been unfeasible given the nature of the interventions; whilst the trial statistician was blind to allocations. Men were randomised at the trial

For peer review only

baseline appointment, where they were provided with all information related to their intervention group in person by the research nurse.

Interventions

Men were randomised to both a physical activity intervention (two levels; brisk walking v control) and a nutrition intervention (three levels; modified nutrition group v lycopene v control). Men in the physical activity intervention group were instructed to walk at a brisk pace for 30 minutes on at least 5 days a week in addition to their usual physical activity, whilst control group men were asked to continue with their usual physical activity. Men in the plant-based diet group (modified nutrition) were instructed to eat as many portions of fruit and vegetables as possible a day, aiming for at least 5 daily portions (printed instructions explained how to measure a portion). In addition, men in the plant-based diet group were asked to reduce their dairy milk intake as much as possible and to use a non-dairy alternative, for example soy, almond or rice milk as often as possible. Men in the lycopene group were asked to consume one 10mg lycopene capsule daily (Holland and Barrett, a UK supplier of nutritional supplements, supplied to the men and paid for by the trial). Men in the nutrition control group were asked to continue with their usual diet. Control men who asked for physical activity or nutrition advice were provided with publicly available information. Instructions on how to perform the interventions were administered by the research nurse. All men were asked to follow their allocated interventions for six months and were contacted at one, two, five, eight, 13, 15 and 18 weeks post-randomisation. The method of contact was chosen by the man: text message, emails, phone call or post. Content of the communication was structured and consistent, regardless of the method of delivery, to ensure similar contact and information provision across participants. These communications contained recipe ideas (plant based diet group) and theory developed motivational messages to encourage continuation of the trial-group intervention (17).

Data Collection

For peer review only

For peer review only - http://bmjopen.bmj.com/site/about/guidelines.xhtml

Men attended the hospital for a research nurse appointment at trial baseline (randomisation), three and six months and completed patient-reported outcome measures (PROMs) (paper questionnaires). If the man was unable to attend the hospital, the questionnaire was sent in the post. Questionnaires analysed here to illustrate the impact of adherence were the Recent Physical Activity Questionnaire (RPAQ) (18) and Food Frequency Questionnaire (FFQ) (19) whilst the other PROMs are detailed elsewhere (17). At baseline, men reported socio-demographic information (level of education, marital status, occupation and ethnicity), and family medical history of cancer. At baseline, three and six months, smoking and alcohol use, and nurse-measured anthropometry, were recorded. Non-fasting blood samples were collected at baseline and six months. All men completed a paper daily monitoring form for the six-month duration logging daily step count, additionally; the physical activity intervention group recorded the duration of brisk walking in minutes; the plant-based diet nutrition group recorded the daily number of portions of fruit or vegetables consumed and the amount of dairy milk replaced by non-dairy alternatives (all / some / none); whilst the lycopene supplementation group recorded whether they had taken the lycopene supplement (yes / no).

Public and Patient Involvement (PPI)

A prostate cancer PPI group was involved from the early protocol development stages. The PPI group contributed to discussions regarding intervention content and length, reviewed trial documentations, such as consent forms and participant information sheets, revised intervention instructions to improve readability and completed questionnaires to assess participant burden.

Sample Size Calculation

The sample size calculation, described previously (17), identified a required minimum sample size of 75 men to estimate an adherence rate (the primary outcome, see below) of 75% with a confidence

For peer review only

interval of ± 5 % (90% power, p=0.05) in each group. We aimed to recruit at least 100 men to the cohort study, anticipating an 80% randomisation rate.

Statistical Analysis

Analyses included all participants with measurements for the primary outcomes and trial baseline values at randomisation in an intention to treat analysis. All statistical analyses used Stata 14 (20).

Primary outcomes

The primary outcomes were: i) the randomisation rate (the number of men agreeing to be randomised into the RCT divided by the total number of men in the cohort) and ii) adherence to the man's allocated intervention at six months following randomisation. Our intended adherence rate was 75% or above. Adherence was based on the four weeks prior to the participants' latest entry in their daily monitoring form. Specifically, for each intervention; to assess adherence to the plant-based diet we analysed the proportion of days that the men reported they consumed five or more portions of fruit or vegetables, and the proportion of days they replaced all or some of their dairy milk with non-dairy alternative. Adherence to the lycopene intervention was based on the proportion of days that the men reported they took the lycopene supplement. The threshold for acceptable adherence was 90%, which equates to adhering to the fruit and vegetable or lycopene interventions on 26 out of a possible 28 days. Adherence to the physical activity intervention was based on number of days of doing 30 or more minutes of self-reported brisk walking. Across the 28 days over which adherence was monitored, men were considered to have adhered 100% to the physical activity intervention if they did 30+ minutes of walking on at least 20 days, while 90% adherence was defined as doing 30+ minutes of walking on 18 of the 28 days.

Secondary outcomes

For peer review only

For peer review only - http://bmjopen.bmj.com/site/about/guidelines.xhtml

PrEvENT secondary outcomes were trial retention, blood biomarkers of fruit and vegetable consumption, self-reported food consumption (measured using the FFQ), self-reported physical activity (measured using the RPAQ), trial tolerability (measured by qualitative interviews and adverse events); these outcomes are reported here. Other secondary outcomes were change in prostate specific antigen and insulin-like growth factor, change in urinary symptoms, psychological factors, health beliefs, quality of life, fatigue, general health, accelerometer and pedometer data and lifestyle behaviours (smoking and drinking). These latter secondary outcomes are not reported here.

As secondary measures of adherence in the dietary interventions, serum concentrations of lutein, zeaxanthin, β-cryptoxanthin, α-carotene, β-carotene and lycopene were measured by reverse-phase high-performance liquid chromatography (HPLC) (21). Assays were standardised against appropriate National Institute of Standards and Technology reference materials (22). These assays are also externally quality assured by participation in the French Society for Vitamins and Biofactors quality assurance scheme (23).

Mean portions of fruit and vegetables consumed per day were calculated from the FFQ. Physical activity energy expenditure was derived from the RPAQ. The effects of the nutrition and physical activity interventions on fruit and vegetable consumption and energy expenditure, respectively, were assessed using multivariable linear regression. As pre-specified, we assumed no interaction between the interventions and tested this through repeating the multivariable linear regression analyses with an interaction term between the interventions, and application of the Wald test with the null hypothesis that the interaction terms were zero. We also compared the consumption of non-dairy milk recorded in the FFQ (categorised into 'Dairy': full cream, semi skimmed, skimmed or dried milk; and 'Non-dairy': soya, other) and blood biomarkers of fruit and vegetable consumption (Lutein, Zeaxanthin, Beta cryptoxanthin, Alpha carotene, Beta carotene, Lycopene) between trial groups using chi-square tests for categorical outcomes and Kruskal-Wallis tests for continuous

For peer review only - http://bmjopen.bmj.com/site/about/guidelines.xhtml

outcomes respectively. A Bonferroni correction was applied to P values (multiplied by two) for post-hoc pairwise comparisons between the nutrition groups (no comparison was made between the lycopene and plant-based interventions) calculated using pairwise chi-square tests for categorical outcomes and Wilcoxon rank-sum tests for continuous outcomes. Intervention tolerability was assessed via adverse events and post-trial qualitative interviews (reported elsewhere).

Exploratory outcomes

In order to describe change in adherence from the beginning to the end of the trial period adherence was also calculated for the first four weeks of daily data monitoring forms after the participants earliest entry. We also calculated weekly adherence to the interventions from one to 26 weeks and overlaid the post-randomisation contact reminders.

RESULTS

Study Recruitment, Participant Characteristics, Randomisation and Follow-up

Between August 2014 and May 2016, 292 eligible men scheduled for radical prostatectomy were invited to participate and of these, 108 (37.0 %) entered the cohort study (Figure 1). The most common reasons for not enrolling were: distance to travel (n=75), not being contactable (n=58), or uninterested in taking part (n=28). Of the 108 men, 96 completed cohort follow up, a further 9 were no longer eligible for the RCT, thus 87 were invited into the RCT and 81 (93.1%, 95% CI 85.8% to 96.8%) consented to be randomised, a trial primary endpoint.

INSERT FIGURE 1: CONSORT diagram

Men were randomised on average around 8 weeks after surgery (range: 4 weeks (31 days) to 15 weeks (108 days)). The men were on average 64.0 years of age (sd = 6.3 years), with 88% (n = 68) self-reporting as white British and with a median pre-operative prostate specific antigen of 8.7 ng/ml

For peer review only - http://bmjopen.bmj.com/site/about/guidelines.xhtml

(inter-quartile range IQR: 5.9 – 12.3). Almost all men were former or never smokers (97.4%, n = 75), and 20% (n = 16) self-reported as consuming more than 14 units of alcohol per week. In general, baseline characteristics were similarly distributed between trial groups (Table 1). Seventy-five of the 81 men completed the trial (92.6 %), three were lost to follow-up and three withdrew. Those who withdrew were for reasons unrelated to the interventions. We present the results of the 75 men who completed the trial.

INSERT TABLE 1: Participant characteristics, baseline measures and clinical data by intervention group

Primary Outcomes:

Adherence to interventions by self-reported daily records

Adherence for at least 90% of the time was 40.0% (95% CI 23.4% to 59.3%, n = 10 of 25) in the plant-based diet group for the fruit and vegetable component and 72.0% (95% CI 52.4% to 85.7%, n = 18 of 25) for the dairy milk component. In the lycopene group, 78.6% (95% CI 60.5% to 89.8%, n = 22 of 28) of men adhered at least 90% of the time. Adherence to the physical activity intervention was 53.8% (95% CI 38.6% to 68.4%, n = 21 of 39) at least 90% of the time (Table 2). Weekly adherence to the interventions appeared stable for most of the intervention period, with a potential drop-off after week 22 in the lycopene and plant based diet arms of the nutrition interventions (Figure 2). Post-randomisation reminders (weeks 1, 2, 5, 8, 13 and 18) did not appear to lead to changes in adherence to the interventions.

INSERT TABLE 2: Adherence to the interventions by self-reported daily monitoring

For peer review only

INSERT FIGURE 2: Weekly adherence to the intervention from 0-26 weeks for participants randomised to the lycopene (a) or plant based diet (b) arms of the nutrition intervention, and physical activity intervention arm (c).[1]

Secondary outcomes:

Effects of the interventions on self-reported diet and physical activity PROMs

Those men in the plant-based diet intervention group reported consuming a mean of 9.9 portions (sd = 4.7 portions) of fruit and vegetables a day based on the FFQ PROMs. This was almost four portions (adjusted difference = 3.7, 95% CI 1.1 to 6.4 portions/day) more than those in the control group (mean = 6.2, sd = 3.8 portions) (P=0.007). Those in the lycopene intervention groups reported consuming a mean of 8.2 portions per day (sd = 4.8 portions), which was 2.1 portions (95% CI -0.5 to 4.7) more per day than the control group (p=0.12) (Table 3). Consumption of non-dairy milk was highest in the plant-based diet intervention group (78.3%, 95% CI 58.1% to 90.3%, n=18 of 23), and was very low otherwise (7.7%, 95% CI 2.1% to 24.1%, 2 of 26; and 0%, 95% CI 0.0% to 16.1%, 0 of 20 for the lycopene intervention and control groups respectively, P<0.001) (Table 3).

INSERT TABLE 3: Effects of interventions on fruit and vegetable consumption, non-dairy milk consumption and energy expenditure at six months

Those men in the physical activity intervention and control groups reported very similar mean energy expenditure based on the PROMs data (mean = 39.7 kj/kg/d, sd = 26.3 kj/kg/d; and mean = 40.8 kj/kg/d, sd = 28.6 kj/kg/d, respectively) (adjusted difference = -0.8 kj/kg/d, 95% CI -13.6 to 11.9 kj/kg/d, p=0.90) (Table 3).

There was no evidence of an interaction between the nutrition and physical activity interventions (F=0.65, P=0.63) so the data are not shown here.

---

[1] Note: Dashed vertical lines represent timepoints (weeks 1, 2, 8, 13, 15, 18) at which reminders were sent to participants

For peer review only - http://bmjopen.bmj.com/site/about/guidelines.xhtml

Effects of the nutrition intervention on blood biomarkers of fruit and vegetable consumption

Lutein (P = 0.07), beta cryptoxanthin (P = 0.07), beta carotene (P = 0.09) and lycopene (P = 0.07) were higher in the plant-based intervention group than the control group. There was no difference in lycopene between the lycopene intervention group compared with the control group (P=0.64). No other differences were found between the blood biomarkers (Table 4).

INSERT TABLE 4: Effects of interventions on blood biomarkers and non-dairy milk consumption at six months

Intervention tolerability

Nine adverse events, self-reported by participants, were classed as unrelated to the interventions and included fever, alcohol withdrawal symptoms, urinary symptoms, diverticulitis, pneumonia, *Campylobacter* infection and hernia repair procedure. Three adverse events were considered as "possibly related to the interventions", these were indigestion, abdominal bloating and knee pain. Data from qualitative interviews reported elsewhere also suggested that men found all the interventions to be acceptable, easy to accommodate and well tolerated (24).

Exploratory analysis

Change in adherence from the beginning to the end of the trial period was examined. In the plant-based group there was an increase in adherence at 6 months compared to the start of the intervention, both for the fruit and vegetable consumption which increased from 20.0% (95% CI 8.9% to 39.1%, n =5 of 25) and the reduced dairy consumption, which increased from 60.0% (95% CI 40.7% to 76.6%, n = 15). Adherence in the lycopene group fell slightly from 89.3% (95% CI 72.8% to 96.3%, n = 25 of 28) within the first four weeks and in the physical activity group which fell slightly

For peer review only

For peer review only - http://bmjopen.bmj.com/site/about/guidelines.xhtml

from 59.0% (95% CI 43.4% to 72.9%, n = 23 of 39) during the first four weeks to reported adherence at 6-month follow up (Table 5).

INSERT TABLE 5: Exploratory data - adherence to the interventions by self-reported daily monitoring (first four weeks)

DISCUSSION

PrEvENT recruited 37.0% of eligible men into the cohort and randomised 93.1% of men from the cohort into the RCT. This is higher than our anticipated randomisation rate of 80%. Forty percent of men in the plant-based diet group adhered to the fruit and vegetable component of the intervention at six months, with more than 70% adhering to the dairy replacement component. Almost 80% of men in the lycopene group adhered to the intervention at six months and just under 54% of men adhered to the physical activity intervention at six months. Those in the plant-based diet consumed 9.9 portions of fruit and vegetables per day, which was more than the nutrition control (6.4 portions); as did the lycopene group, at 8.2 portions per day. Over 90% completed 6-month follow up.

Adherence for both elements of the plant-based intervention increased from the first month of the intervention to the last month. This may reflect the men's need to develop new shopping, eating and social behaviours to incorporate the increased fruit and vegetable consumption as well as the reduction in dairy milk. It should be noted that our definition of adherence was strict, expecting participants to follow the intervention more than 90% of the time. Previous diet and physical activity interventions in cancer populations have reported higher adherence rates of around 70%. However, in these instances the interventions were either less intensive or definitions of adherence were not as strict as the current study (25-27).

For peer review only - http://bmjopen.bmj.com/site/about/guidelines.xhtml

The findings from our blood biomarker analysis further contribute to the conclusion of acceptable adherence, with higher levels of lutein and beta cryptoxanthin evident in the plant-based population at six months, compared to the other groups. Both carotenoids are found in fruit and vegetables, for example dark leafy greens, oranges, red peppers and carrots, thus implying higher consumption of certain fruits and vegetables in the plant-based diet group compared to the other groups. Almost 80% of the plant-based group self-reported consuming non-dairy milk products at six months compared to 8% of the lycopene group and none of the control group, suggestive of high compliance with that intervention.

Adherence to lycopene supplements was high at almost 80% at six months, a slight drop from the first four weeks of the trial where almost 90% adherence was reported. This may have been down to the simplicity of taking a lycopene supplement, where the majority of men were able to easily incorporate the capsule into their daily routine (24). It should be noted that despite excellent adherence to the lycopene supplementation, no differences in lycopene levels were reported in the blood biomarkers between men in the lycopene and other groups. Assuming self-reported lycopene supplementation consumption was correct, this finding may suggest the supplement dosage of 10mg per day was not high enough. This should be considered when designing future lycopene interventions. Our high adherence rates, however, confirm the feasibility of this population taking a daily lycopene supplement, which was an outcome of the trial.

The plant-based adherence rates were lower than the anticipated 75%, although adherence to the reduced dairy milk component were higher, almost reaching this target; the lycopene adherence rates were higher than the proposed 75%. Almost 54% of participants adhered to the physical activity intervention at six months, slightly lower than the 59% adhering within the first four weeks of the trial.

For peer review only

Overall, these randomisation and adherence rates suggest that men were willing to take part and generally followed the intervention instructions well. It should be noted that as a feasibility trial we were not concerned with the clinical relevance of these changes, but whether participants would make, and adhere to, the changes for the duration of the trial.

The finding that participants in the plant-based diet intervention group consumed four additional daily portions of fruit or vegetables than the control group is substantial from a public health perspective. The lycopene intervention group also consumed a significantly larger amount of fruit and vegetables than the control group, although less than the plant-based diet participants. This may have been for one of two reasons. Firstly, the information sheet explained that lycopene is found in red food products such as tomatoes and grapefruit, thus the lycopene group may have increased fruit and vegetable consumption for this reason, as well as taking the supplement. Secondly, it may have been the case that being in an active nutrition group, regardless of the specific instructions, increased participants awareness about their nutrition, which may have resulted in them consuming more fruit and vegetables (12).

Overall consumption of fruit and vegetables of all participants was reported to be high. Seven or eight portions per day were reported at baseline, increasing to eight to ten portions at six months (nutrition control was slightly lower at six portions). This is proportionately higher than the national average. In 2013/2014, 27% of those aged 19 to 64 years and 35% of those aged 65 years and over met the 5-A-Day recommendation (28, 29). The average consumption for these age groups were 4 and 4.2 portions of fruit and vegetables per day, respectively (29). There may be a number of explanations for this. Due to the information in the public domain surrounding prostate cancer, which includes general advice to eat a healthy balanced diet, many of the men may have increased their fruit and vegetable consumption at time of diagnosis (11, 12). Alternatively, as all participants had already participated in the cohort, which asked questions about their diet, this may have made

For peer review only - http://bmjopen.bmj.com/site/about/guidelines.xhtml

For peer review only

them consider their diet prior to entering the RCT and thus increased consumption (12). Finally, there may have been some over-reporting of fruit and vegetable consumption, either due to social desirability, inconsistent recall or as a result of previously reported issues with the FFQ (30). This may explain the disparity between the reported high consumption of fruit and vegetables, but lower adherence to the plant-based intervention.

There were no differences in levels of energy expenditure reported between the physical activity intervention group and control group. It may have been the case that no differences existed; or that the RPAQ was not sensitive enough to detect differences. Alternatively, it may have been that the physical activity intervention was not vigorous enough to warrant a difference in score between the physical activity intervention groups. This is something to be noted for future physical activity interventions, with the possibility of increasing the intensity of the intervention, with the potential to influence weight management.

Limitations:

There are some limitations. Except for the statistician, all researchers and participants were unblinded. Men were provided with information on the specifics of all trial groups and therefore there may have been cross over in behaviour, potentially diluting results. However, blinding was not feasible given the nature of the intervention. The population lacked heterogeneity, particularly with regards to ethnicity, possibly limiting the generalisation of results. Many of the key variables were assessed via PROMs, potentially causing responder bias. However, we also report objective biochemical measures which supported the inference of good adherence, particularly within the plant-based group. It should be noted that the two key reasons for not enrolling were having too far to travel to attend research clinics or no response to the invitation being received. If these two barriers were removed, then our data suggests that the cohort recruitment could potentially have increased to 51%.

For peer review only - http://bmjopen.bmj.com/site/about/guidelines.xhtml

Conclusion

The PrEvENT interventions were feasible as evidenced by high randomisation rates, high retention rates and excellent tolerability. Men demonstrated good adherence to all interventions, particularly the lycopene supplement, brisk walking and the reduced dairy milk element of the plant-based diet interventions. Positive effects on fruit and vegetable consumption were seen in the plant-based diet and lycopene groups. The development of a definitive RCT should consider: travel distance to research clinics; increasing the intensity of the physical activity intervention, and dosage of the lycopene supplementation; and possibly reducing the minimum criteria for adherence.

ADDITIONAL INFORMATION

Ethics approval and consent to participate

The trial was performed in accordance with the Declaration of Helsinki, and the ethical principles of the International Conference on Harmonisation – Good Clinical Practice. All study procedures were approved by the National Research Ethics Committee South West – Cornwall and Plymouth (REC reference number 14/SW/0056). All men provided fully informed written consent at both the cohort and RCT recruitment stages. The trial was registered with the ISRCTN registry on 17th November 2014 (ISRCTN99048944).

Availability of data and materials

The datasets generated and analysed during the current study are available from the corresponding author on reasonable request.

Authorship

LHM conceived and designed the work, acquired and interpreted data and drafted the manuscript. CP interpreted data and drafted the manuscript. ES and LAR acquired data. CMP, JMPH, ER, AK, AB

For peer review only

For peer review only - http://bmjopen.bmj.com/site/about/guidelines.xhtml

and RP designed the work. CS, LJ, KMB and AF acquired data. JVW and SG interpreted data. JO and PA designed the work. JAL and RMM conceived the work, designed the work and interpreted data. All authors revised the manuscript, approved the final version of the manuscript and agree to be accountable for all aspects of the work.

Acknowledgements

The authors wish to acknowledge Mr Stu Toms for his development of the trial database and data management support throughout.

Funding

This study was supported by the NIHR Biomedical Research Centre at University Hospitals Bristol NHS Foundation Trust and the University of Bristol. The views expressed in this publication are those of the authors and not necessarily those of the NHS, the National Institute for Health Research or the Department of Health.

The Bristol Randomised Trials Collaboration (BRTC) receives National Institute for Health Research CTU Support Funding. The BRTC is a UK Clinical Research Collaboration registered CTU and a member of the NCRI Cancer Clinical Trials Group.

The views expressed are those of the authors and not necessarily those of the NHS, the NIHR, BRTC or the Department of Health.

Conflict of interest

The authors declare no conflict of interest.

For peer review only

For peer review only - http://bmjopen.bmj.com/site/about/guidelines.xhtml

References

1. Cancer Research UK. Prostate cancer statistics 2019 [Available from: http://www.cancerresearchuk.org/health-professional/cancer-statistics/statistics-by-cancer-type/prostate-cancer#heading-Zero].
2. Hackshaw-McGeagh LE, Perry RE, Leach VA, Qandil S, Jeffreys M, Martin RM. A systematic review of dietary, nutritional and physical activity interventions for the prevention of prostate cancer progression and mortality. Cancer Causes Control. 2015;26:1521-50.
3. World Cancer Research Fund. Continuous Update Project Report: Diet, nutrition, physical activity and prostate cancer. 2014.
4. Chan JM, Holick CN, Leitzmann MF, Rimm EB, Willett WC, Stampfer MJ. Diet after diagnosis and the risk of prostate cancer progression, recurrence, and death (United States). Cancer Causes Control. 2006;17:199-208.
5. Neill MG, Fleshner NE. An update on chemoprevention strategies in prostate cancer for 2006. Curr Opin Urol. 2006;16:132-7.
6. Downer MK, Batista JL, Mucci LA, Stampfer MJ, Epstein MM, Hakansson N, et al. Dairy intake in relation to prostate cancer survival. Int J Cancer. 2017;140(9):2060-9.
7. Kruk J, Aboul-Enein H. What Are the Links of Prostate Cancer with Physical Activity and Nutrition? : A Systematic Review Article. Iranian Journal of Public Health. 2016;45(12):1558-67.
8. Segal R, Zwaal C, Green E, Tomasone JR, Loblaw A, Petrella T, et al. Exercise for people with cancer: a systematic review. Curr Oncol. 2017;24(4):e290-e315.
9. Physical Activity Guidelines Advisory Committee. Physical Activity Guidelines Advisory Committee Report, 2008. 2008.
10. Studenski S PS, Patel K, Rosano C, Faulkner K, Inzitari M, Brach J, Chandler J, Cawthon P, Connor EB, Nevitt M, Visser M, Kritchevsky S, Badinelli S, Harris T, Newman AB, Cauley J, Ferrucci L, Guralnik J. Gait speed and survival in older adults. JAMA. 2011;305(1):50 - 8.
11. Hackshaw-McGeagh LE, Penfold CM, Walsh E, Donovan JL, Hamdy FC, Neal DE, et al. Physical activity, alcohol consumption, BMI and smoking status before and after prostate cancer diagnosis in the ProtecT trial: opportunities for lifestyle modification. Int J Cancer. 2015;137(6):1509-15.
12. Avery KNL, Donovan JL, Gilbert R, Davis M, Emmett P, Down L, et al. Men with prostate cancer make positive dietary changes following diagnosis and treatment. Cancer Causes & Control. 2013;24(6):1119-28.
13. Karlsen RV, Bidstrup PE, Christensen J, Larsen SB, Tjonneland A, Dalton SO, et al. Men with cancer change their health behaviour: a prospective study from the Danish diet, cancer and health study. Br J Cancer. 2012;107(1):201-6.
14. Sutton E, Hackshaw-McGeagh LE, Aning J, Bahl A, Koupparis A, Persad R, et al. The provision of dietary and physical activity advice for men diagnosed with prostate cancer: a qualitative study of the experiences and views of health care professionals, patients and partners. Cancer Causes & Control. 2017;28(4):319-29.
15. Demark-Wahnefried W, Aziz NM, Rowland JH, Pinto BM. Riding the crest of the teachable moment: promoting long-term health after the diagnosis of cancer. J Clin Oncol. 2005;23(24):5814-30.
16. Williams K, Steptoe A, Wardle J. Is a cancer diagnosis a trigger for health behaviour change? Findings from a prospective, population-based study. Br J Cancer. 2013;108(11):2407-12.
17. Hackshaw-McGeagh L, Lane JA, Persad R, Gillatt D, Holly JM, Koupparis A, et al. Prostate cancer - evidence of exercise and nutrition trial (PrEvENT): study protocol for a randomised controlled feasibility trial. Trials. 2016;17(1):123.
18. Besson H, Brage S, Jakes RW, Ekelund U, Wareham NJ. Estimating physical activity energy expenditure, sedentary time, and physical activity intensity by self-report in adults. The American journal of clinical nutrition. 2010;91(1):106-14.

For peer review only

562 19. Willett WC, Sampson L, Stampfer MJ, Rosner B, Bain C, Witschi J. Reproducibility and validity of a semiquantitative food frequency questionnaire. Am J Epidemiol. 1985;122.
564 20. StataCorp. Stata Statistical Software: Release 14. College Station, TX: StataCorp LP; 2015.
565 21. Craft N, Wise S, H. Soares J. Optimisation of an isocratic high performance liquid chromatography separation of carotenoids. Journal of Chromatography 1992;589(1-2):171-6.
567 22. National Institute of Standards and Technology. Certification of Standard Reference Material® 968f Fat-Soluble Vitamins in Frozen Human Serum. 2017.
569 23. Societe Francaise Vitamines and Biofacteurs. 2011 Quality Assurance Program: Societe Francaise Vitamines and Biofacteurs; 2011 [Available from: http://www.sfvb.org/pages/aquality.htm.
572 24. Shingler E, Hackshaw-McGeagh L, Robles L, Persad R, Koupparis A, Rowe E, et al. The feasibility of the Prostate cancer: Evidence of Exercise and Nutrition Trial (PrEvENT) dietary and physical activity modifications: a qualitative study. Trials. 2017;18(1):106.
575 25. Koutoukidis DA, Beeken RJ, Manchanda R, Michalopoulou M, Burnell M, Knobf MT, et al. Recruitment, adherence, and retention of endometrial cancer survivors in a behavioural lifestyle programme: the Diet and Exercise in Uterine Cancer Survivors (DEUS) parallel randomised pilot trial. BMJ Open. 2017;7(10).
579 26. Demark-Wahnefried W, Clipp EC, Lipkus IM, Lobach D, Snyder DC, Sloane R, et al. Main Outcomes of the FRESH START Trial: A Sequentially Tailored, Diet and Exercise Mailed Print Intervention Among Breast and Prostate Cancer Survivors. Journal of Clinical Oncology. 2007;25(19):2709-18.
583 27. James EL, Stacey FG, Chapman K, Boyes AW, Burrows T, Girgis A, et al. Impact of a nutrition and physical activity intervention (ENRICH: Exercise and Nutrition Routine Improving Cancer Health) on health behaviors of cancer survivors and carers: a pragmatic randomized controlled trial. BMC Cancer. 2015;15(1):710.
587 28. Department of Health. 5-A-Day [Available from: http://www.nhs.uk/Livewell/5ADAY/Pages/5ADAYhome.aspx.
589 29. Public Health England. National Diet and Nutrition Survey Results from Years 5 and 6 (combined) of the Rolling Programme (2012/2013 – 2013/2014). 2016.
591 30. Cade JE, Burley VJ, Warm DL, Thompson RL, Margetts BM. Food-frequency questionnaires: a review of their design, validation and utilisation. Nutrition Research Reviews. 2007;17(1):5-22.

For peer review only - http://bmjopen.bmj.com/site/about/guidelines.xhtml

Table 1: Participant characteristics, baseline measures and clinical data by intervention groups

	n (% of participants in group)				
	Nutrition (n=81)			Physical activity (n=81)	
	Lycopene intervention (n=28, 34.6%)	Plant based diet intervention (n=27, 33.3%)	Nutrition control (n=26, 32.1%)	PA control (n=39, 48.1%)	PA intervention (n=42, 51.9%)
**Participant characteristics**					
**Age (years)***	62.9 (7.7)	64.6 (5.7)	64.7 (5.5)	62.5 (6.9)	65.5 (5.5)
**Ethnicity**					
White British	23 (88.5)	23 (85.2)	22 (91.7)	33 (86.8)	35 (89.7)
**Highest education level^**					
Standard or less (e.g. o-levels, GCSE)	7 (26.9)	12 (44.4)	14 (60.9)	20 (52.6)	13 (34.2)
Further education (e.g. A-levels, HND, University degree)	19 (73.1)	15 (55.6)	9 (39.1)	18 (47.4)	25 (65.8)
**Marital status**					
Married / in a relationship	23 (88.5)	22 (81.5)	22 (95.7)	33 (86.8)	34 (89.5)
**Father had prostate cancer**					
Yes	11 (44.0)	2 (7.4)	1 (4.3)	8 (21.6)	6 (15.8)
**Smoking status**					
Current	0 (0.0)	0 (0.0)	2 (7.7)	1 (2.7)	1 (2.5)
Former	9 (37.5)	13 (48.1)	16 (61.5)	21 (56.8)	17 (42.5)
Never	15 (62.5)	14 (51.9)	8 (30.8)	15 (40.5)	22 (55.0)
**Alcohol consumption (units/week)$^+$**	1.0 (0.0; 7.0)	4.0 (0.0; 12.0)	4.5 (0.0; 22.0)	2.0 (0.0; 7.0)	4.0 (0.0; 13.0)
**Alcohol consumption (categorised)$^\#$**					
Non-drinker (0 units/week)	14 (50.0)	9 (33.3)	8 (30.8)	17 (43.6)	14 (33.3)
≤14 units/week	11 (39.3)	14 (51.9)	9 (34.6)	15 (38.5)	19 (45.2)
>14 units/week	3 (10.7)	4 (14.8)	9 (34.6)	7 (17.9)	9 (21.4)
**Body mass index (kg/m^2)***	26.2 (3.6)	26.7 (3.6)	26.8 (3.1)	26.6 (3.5)	26.6 (3.3)
**Baseline measures and clinical data**					
**Pre-surgical Prostate specific antigen (ng/mL)$^+$**	7.8 (5.2; 11.0)	8.4 (5.7; 9.6)	10.6 (8.4; 15.0)	8.4 (5.7; 11.0)	8.9 (6.4; 12.7)

For peer review only - http://bmjopen.bmj.com/site/about/guidelines.xhtml

**Baseline fruit and vegetable consumption (portions/day)***	7.5 (4.7)	8.5 (5.7)	7.3 (2.4)	8.2 (5.2)	7.4 (3.7)
**Baseline energy expenditure (kJ/kg/d)***	27.2 (32.9)	22.8 (13.9)	27.4 (21.6)	23.1 (23.0)	28.3 (25.0)
**Type of milk consumed most often**					
Dairy	22 (88.0)	22 (84.6)	24 (96.0)	30 (83.3)	38 (95.0)
Non-dairy	3 (12.0)	4 (15.4)	1 (4.0)	6 (16.7)	2 (5.0)
**Lutein - umol/l^{+}**	0.36 (0.28; 0.55)	0.40 (0.32; 0.52)	0.35 (0.20; 0.46)	0.35 (0.28; 0.52)	0.38 (0.28; 0.51)
**Zeaxanthin - umol/l^{+}**	0.11 (0.09; 0.16)	0.10 (0.09; 0.12)	0.10 (0.06; 0.13)	0.11 (0.08; 0.12)	0.10 (0.08; 0.14)
**Beta cryptoxanthin - umol/l^{+}**	0.22 (0.16; 0.36)	0.25 (0.15; 0.36)	0.19 (0.11; 0.39)	0.22 (0.14; 0.32)	0.24 (0.14; 0.40)
**Alpha carotene - umol/l^{+}**	0.07 (0.05; 0.09)	0.08 (0.06; 0.10)	0.07 (0.05; 0.08)	0.07 (0.05; 0.08)	0.07 (0.06; 0.09)
**Beta carotene - umol/l^{+}**	0.64 (0.46; 1.06)	0.72 (0.50; 1.16)	0.56 (0.47; 0.84)	0.66 (0.47; 0.89)	0.64 (0.48; 1.07)
**Lycopene - umol/l^{+}**	1.35 (1.03; 1.69)	1.24 (0.92; 1.44)	1.20 (0.90; 1.59)	1.23 (0.92; 1.47)	1.29 (1.04; 1.62)

* Values are mean (s.d) $^{+}$ Values are median (25%; 75%) ^ O-level, GCSE: national school examinations at age 16 years. A-level: national school examinations at age 18 years. HND (Higher National Diploma) is a work-related course provided by higher and further education colleges in the UK. A full-time HND takes two years to complete, and generally is the equivalent to two years at university # Non-drinker: 0 units/week, Moderate: >0 & ≤14 units/week, Hazardous: >14 & ≤50 units/week, Harmful: >50 units/week

Table 2: Adherence to the interventions by self-reported daily monitoring[2]

[2] Adherence was based on the four weeks prior to the participants' latest entry in their daily monitoring form. The threshold for acceptable adherence was 90%, which equates to adhering to the fruit and vegetable or lycopene interventions on 26 out of a possible 28 days. Adherence to the physical activity

For peer review only

	Adherence to lycopene intervention[1]	Adherence to plant based diet intervention[1]		Adherence to physical activity intervention[1]
		Fruit and vegetables	Dairy replacement	
**Physical activity (PA)**				
PA control (N=36)	30.6% (n = 11)	13.9% (n = 5)	30.6% (n = 11)	-
PA intervention (N=39)	28.2% (n = 11)	12.8% (n = 5)	18.0% (n = 7)	53.8% (n = 21)
**Nutrition**				
Lycopene intervention (N=28)	78.6% (n = 22)	-	-	28.6% (n = 8)
Plant based diet intervention (N=25)	-	40.0% (n = 10)	72.0% (n = 18)	28.0% (n = 7)
Nutrition control (N=22)	-	-	-	27.3% (n = 6)

602

intervention equates to doing 30+ minutes of walking on 18 of the 28 days.

For peer review only - http://bmjopen.bmj.com/site/about/guidelines.xhtml

For peer review only

Table 3: Effects of interventions on fruit and vegetable consumption, non-dairy milk consumption and energy expenditure at six months

	Nutrition (n=75)			Physical Activity (n=75)	
	Lycopene intervention (n=28)	Plant based diet intervention (n=25)	Nutrition control (n=22)	Physical activity control (n=36)	Physical activity intervention (n=39)
Fruit and veg (portions/day), mean (sd)	8.2 (4.8)	9.9 (4.7)	6.2 (3.8)	8.3 (4.9)	8.1 (4.5)
Adjusted difference in fruit and vegetable consumption (portions/day) compared to the control group* (95% CI)	2.07 (-0.51-4.65)	3.73 (1.07-6.38)	-	-	-0.20 (-2.37 – 1.98)
P value	0.12[1]	0.007[1]			0.86[1]
Energy expenditure (kJ/kg/d), mean (sd)	41.8 (23.7)	43.1 (32.0)	34.9 (26.1)	40.8 (28.6)	39.7 (26.3)
Adjusted difference in energy expenditure (kJ/kg/d) compared to the control group* (95% CI)	6.91 (-8.77 – 22.6)	8.19 (-7.89 – 24.3)	-	-	-0.84 (-13.6 – 11.9)
P value	0.38[1]	0.31[1]			0.90[1]
**Type of milk consumed most often**					
Dairy, n (%)	24 (92.3)	5 (21.7)	20 (100.0)	22 (66.7)	27 (75.0)
Non-dairy, n (%)	2 (7.7)	18 (78.3)	0 (0.0)	11 (33.3)	9 (25.0)
P-values	*0.41*[3]	*<0.001*[3]	<0.001[2]		0.08[2]

***** **-** fruit and vegetable consumption adjusted for physical activity intervention group, and energy expenditure adjusted for nutrition intervention group

For peer review only

For peer review only - http://bmjopen.bmj.com/site/about/guidelines.xhtml

605 [1] P-values from multivariate linear regression, [2] P-values from Wilcoxon sign-rank tests, [3] P-values from Wilcoxon sign-rank test with Bonferroni correction

For peer review only

For peer review only - http://bmjopen.bmj.com/site/about/guidelines.xhtml

Table 4: Effects of interventions on blood biomarkers at six months

	**Nutrition (n=75)**						**Physical Activity (n=75)**		
	**Lycopene intervention (n = 28) Median (IQR)**	**Plant based diet intervention (n = 25) Median (IQR)**	**Nutrition control (n = 22) Median (IQR)**	**P** [1]	**P** (pairwise comparisons) [2] **Lycopene vs control**	**Plant based vs control**	**Physical Activity intervention (n = 39) Median (IQR)**	**Physical Activity control (n = 36) Median (IQR)**	**P**[1]
**Lycopene** (umol/l)	1.30 (0.96; 1.60)	1.41 (1.12; 1.79)	1.16 (0.83; 1.39)	0.11	0.64	0.07	1.27 (0.95; 1.71)	1.36 (0.97; 1.58)	0.90
**Lutein** (umol/l)	0.37 (0.28; 0.46)	0.49 (0.36; 0.57)	0.36 (0.27; 0.48)	0.05	1.00	0.07	0.40 (0.29; 0.49)	0.38 (0.26; 0.55)	0.56
**Zeaxanthin** (umol/l)	0.09 (0.07; 0.16)	0.12 (0.10; 0.16)	0.10 (0.08; 0.14)	0.26	0.92	0.22	0.12 (0.08; 0.15)	0.11 (0.08; 0.16)	0.89
**Beta cryptoxanthin** (umol/l)	0.22 (0.11; 0.39)	0.30 (0.23; 0.51)	0.23 (0.12; 0.29)	0.06	1.00	0.07	0.24 (0.13; 0.50)	0.23 (0.16; 0.35)	0.44
**Alpha carotene** (umol/l)	0.07 (0.06; 0.09)	0.08 (0.06; 0.13)	0.07 (0.05; 0.09)	0.26	1.00	0.28	0.07 (0.05; 0.10)	0.07 (0.06; 0.09)	0.52
**Beta carotene** (umol/l)	0.59 (0.37; 0.97)	0.81 (0.54; 1.24)	0.57 (0.46; 0.79)	0.11	1.00	0.09	0.73 (0.45; 1.16)	0.64 (0.43; 0.93)	0.47

[1] P-values from Kruskal-Wallis tests unless stated otherwise, [2] P-values from Wilcoxon rank-sum tests, with Bonferroni correction unless stated otherwise

umol/l – micromole per litre.

For peer review only - http://bmjopen.bmj.com/site/about/guidelines.xhtml

610 **Table 5: Exploratory data - adherence to the interventions by self-reported daily monitoring (first four weeks)[3]**

	Adherence to lycopene intervention[2]	Adherence to plant based diet intervention[2]		Adherence to physical activity intervention[2]
		Fruit and vegetables	Dairy replacement	
**Physical activity**				
PA control (N=36)	**33.3% (n = 12)**	**2.8% (n = 1)**	**19.4% (n = 7)**	-
PA intervention (N=39)	**33.3% (n = 13)**	**10.3% (n = 4)**	**20.5% (n = 8)**	**59.0% (n = 23)**
**Nutrition**				
Lycopene intervention (N=28)	**89.3% (n = 25)**	-	-	**39.3% (n = 11)**
Plant based diet intervention (N=25)	-	**20.0% (n = 5)**	**60.0% (n = 15)**	**20.0% (n = 5)**
Nutrition control (N=22)	-	-	-	**31.8% (n = 7)**

[3] Adherence was based on the first four weeks of daily data monitoring forms after the participants earliest entry. The threshold for acceptable adherence was 90%, which equates to adhering to the fruit and vegetable or lycopene interventions on 26 out of a possible 28 days. Adherence to the physical activity intervention equates to doing 30+ minutes of walking on 18 of the 28 days.

For peer review only - http://bmjopen.bmj.com/site/about/guidelines.xhtml

612

For peer review only

For peer review only - http://bmjopen.bmj.com/site/about/guidelines.xhtml

Figure 1: CONSORT diagram

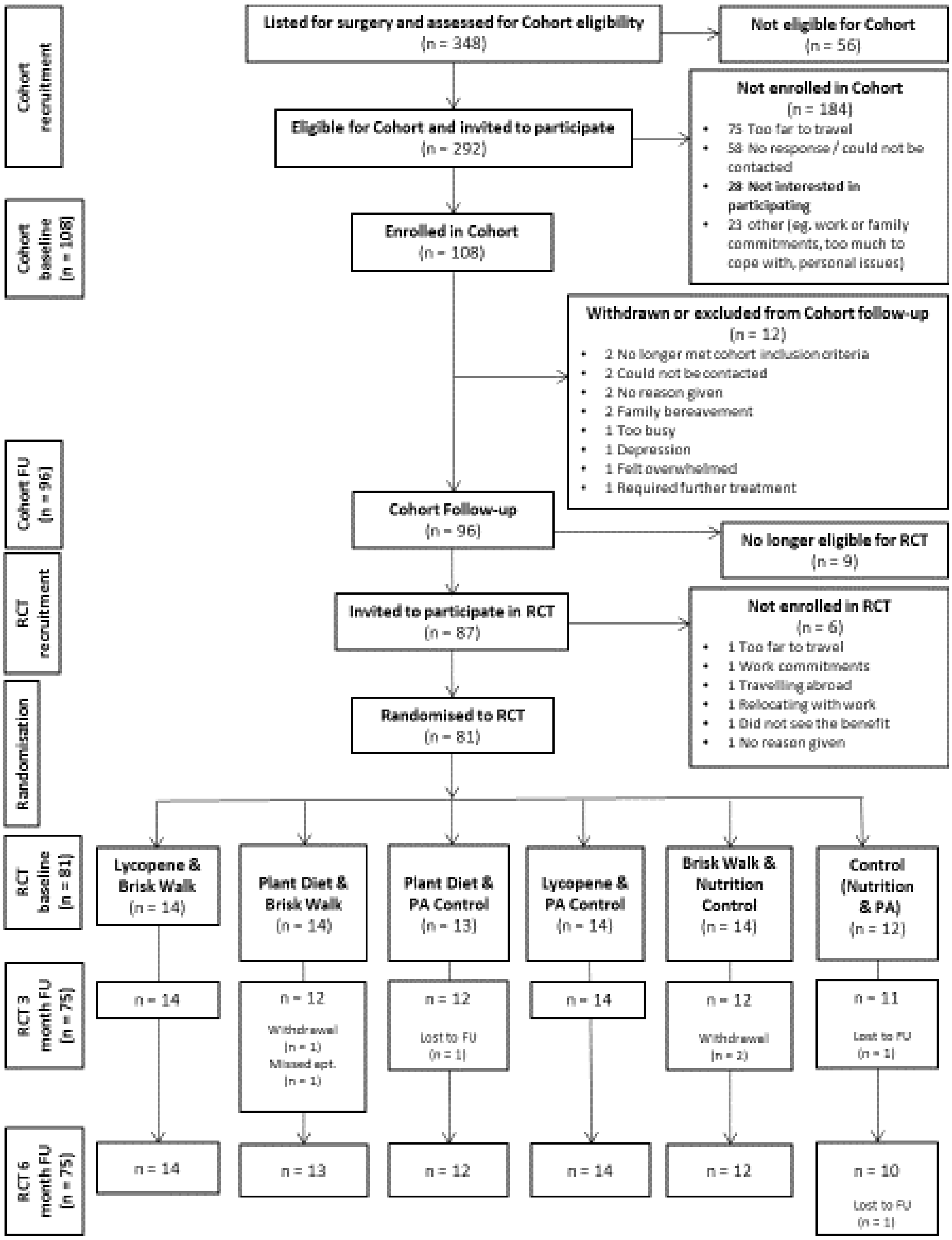

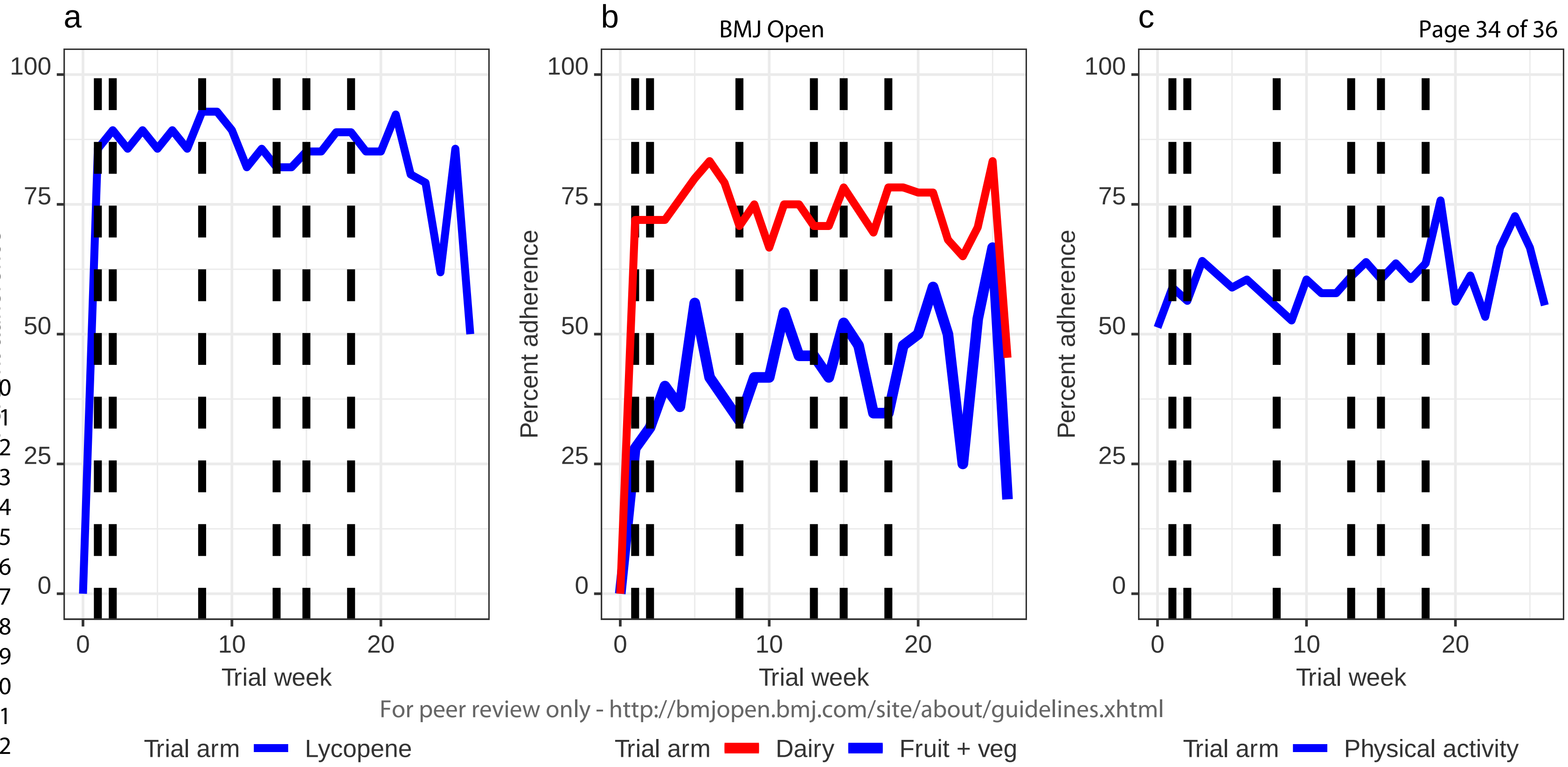
a
Percent adherence
100
75
50
25
0
0
10
20
Trial week
Trial arm
Lycopene
b
Percent adherence
100
75
50
25
0
0
10
20
Trial week
Trial arm
Dairy
Fruit + veg
c
Percent adherence
100
75
50
25
0
0
10
20
Trial week
Trial arm
Physical activity

CONSORT 2010 checklist of information to include when reporting a pilot or feasibility trial*

Section/Topic	Item No	Checklist item	Reported on page No
**Title and abstract**			
	1a	Identification as a pilot or feasibility randomised trial in the title	1
	1b	Structured summary of pilot trial design, methods, results, and conclusions (for specific guidance see CONSORT abstract extension for pilot trials)	3-4
**Introduction**			
Background and objectives	2a	Scientific background and explanation of rationale for future definitive trial, and reasons for randomised pilot trial	5-6
	2b	Specific objectives or research questions for pilot trial	6
**Methods**			
Trial design	3a	Description of pilot trial design (such as parallel, factorial) including allocation ratio	6
	3b	Important changes to methods after pilot trial commencement (such as eligibility criteria), with reasons	Na
Participants	4a	Eligibility criteria for participants	7
	4b	Settings and locations where the data were collected	8-9
	4c	How participants were identified and consented	7-8
Interventions	5	The interventions for each group with sufficient details to allow replication, including how and when they were actually administered	8
Outcomes	6a	Completely defined prespecified assessments or measurements to address each pilot trial objective specified in 2b, including how and when they were assessed	10-11
	6b	Any changes to pilot trial assessments or measurements after the pilot trial commenced, with reasons	Na
	6c	If applicable, prespecified criteria used to judge whether, or how, to proceed with future definitive trial	Na
Sample size	7a	Rationale for numbers in the pilot trial	9
	7b	When applicable, explanation of any interim analyses and stopping guidelines	Na
Randomisation:			
Sequence generation	8a	Method used to generate the random allocation sequence	7
	8b	Type of randomisation(s); details of any restriction (such as blocking and block size)	7
Allocation concealment	9	Mechanism used to implement the random allocation sequence (such as sequentially numbered containers), describing any steps taken to conceal the sequence until interventions were assigned	7

For peer review only

mechanism			
Implementation	10	Who generated the random allocation sequence, who enrolled participants, and who assigned participants to interventions	7
Blinding	11a	If done, who was blinded after assignment to interventions (for example, participants, care providers, those assessing outcomes) and how	7
	11b	If relevant, description of the similarity of interventions	Na
Statistical methods	12	Methods used to address each pilot trial objective whether qualitative or quantitative	10
**Results**			
Participant flow (a diagram is strongly recommended)	13a	For each group, the numbers of participants who were approached and/or assessed for eligibility, randomly assigned, received intended treatment, and were assessed for each objective	Figure 1
	13b	For each group, losses and exclusions after randomisation, together with reasons	Figure 1
Recruitment	14a	Dates defining the periods of recruitment and follow-up	12
	14b	Why the pilot trial ended or was stopped	Na
Baseline data	15	A table showing baseline demographic and clinical characteristics for each group	Table 1
Numbers analysed	16	For each objective, number of participants (denominator) included in each analysis. If relevant, these numbers should be by randomised group	Table 1
Outcomes and estimation	17	For each objective, results including expressions of uncertainty (such as 95% confidence interval) for any estimates. If relevant, these results should be by randomised group	Table 2-5
Ancillary analyses	18	Results of any other analyses performed that could be used to inform the future definitive trial	Table 5
Harms	19	All important harms or unintended effects in each group (for specific guidance see CONSORT for harms)	14
	19a	If relevant, other important unintended consequences	Na
**Discussion**			
Limitations	20	Pilot trial limitations, addressing sources of potential bias and remaining uncertainty about feasibility	19
Generalisability	21	Generalisability (applicability) of pilot trial methods and findings to future definitive trial and other studies	15-19
Interpretation	22	Interpretation consistent with pilot trial objectives and findings, balancing potential benefits and harms, and considering other relevant evidence	15-18
	22a	Implications for progression from pilot to future definitive trial, including any proposed amendments	19
**Other information**			
Registration	23	Registration number for pilot trial and name of trial registry	20
Protocol	24	Where the pilot trial protocol can be accessed, if available	6
Funding	25	Sources of funding and other support (such as supply of drugs), role of funders	20-21
	26	Ethical approval or approval by research review committee, confirmed with reference number	19-20

For peer review only

Citation: Eldridge SM, Chan CL, Campbell MJ, Bond CM, Hopewell S, Thabane L, et al. CONSORT 2010 statement: extension to randomised pilot and feasibility trials. BMJ. 2016;355.
*We strongly recommend reading this statement in conjunction with the CONSORT 2010, extension to randomised pilot and feasibility trials, Explanation and Elaboration for important clarifications on all the items. If relevant, we also recommend reading CONSORT extensions for cluster randomised trials, non-inferiority and equivalence trials, non-pharmacological treatments, herbal interventions, and pragmatic trials. Additional extensions are forthcoming: for those and for up to date references relevant to this checklist, see www.consort-statement.org.

For peer review only
